# Prediction and clinical utility of a contralateral breast cancer risk model

**DOI:** 10.1186/s13058-019-1221-1

**Published:** 2019-12-17

**Authors:** Daniele Giardiello, Ewout W. Steyerberg, Michael Hauptmann, Muriel A. Adank, Delal Akdeniz, Carl Blomqvist, Stig E. Bojesen, Manjeet K. Bolla, Mariël Brinkhuis, Jenny Chang-Claude, Kamila Czene, Peter Devilee, Alison M. Dunning, Douglas F. Easton, Diana M. Eccles, Peter A. Fasching, Jonine Figueroa, Henrik Flyger, Montserrat García-Closas, Lothar Haeberle, Christopher A. Haiman, Per Hall, Ute Hamann, John L. Hopper, Agnes Jager, Anna Jakubowska, Audrey Jung, Renske Keeman, Iris Kramer, Diether Lambrechts, Loic Le Marchand, Annika Lindblom, Jan Lubiński, Mehdi Manoochehri, Luigi Mariani, Heli Nevanlinna, Hester S. A. Oldenburg, Saskia Pelders, Paul D. P. Pharoah, Mitul Shah, Sabine Siesling, Vincent T. H. B. M. Smit, Melissa C. Southey, William J. Tapper, Rob A. E. M. Tollenaar, Alexandra J. van den Broek, Carolien H. M. van Deurzen, Flora E. van Leeuwen, Chantal van Ongeval, Laura J. Van’t Veer, Qin Wang, Camilla Wendt, Pieter J. Westenend, Maartje J. Hooning, Marjanka K. Schmidt

**Affiliations:** 1 Division of Molecular Pathology, The Netherlands Cancer Institute - Antoni van Leeuwenhoek Hospital, Amsterdam, The Netherlands; 2 Department of Biomedical Data Sciences, Leiden University Medical Center, Leiden, The Netherlands; 3 Department of Public Health, Erasmus MC Cancer Institute, Rotterdam, The Netherlands; 4 Institute of Biometry and Registry Research, Brandenburg Medical School, Neuruppin, Germany; 5 Department of Epidemiology and Biostatistics, The Netherlands Cancer Institute - Antoni van Leeuwenhoek Hospital, Amsterdam, The Netherlands; 6 The Netherlands Cancer Institute - Antoni van Leeuwenhoek hospital, Family Cancer Clinic, Amsterdam, The Netherlands; 7 Department of Medical Oncology, Family Cancer Clinic, Erasmus MC Cancer Institute, Rotterdam, The Netherlands; 8 Department of Oncology, Helsinki University Hospital, University of Helsinki, Helsinki, Finland; 9 Department of Oncology, Örebro University Hospital, Örebro, Sweden; 10 Copenhagen General Population Study, Herlev and Gentofte Hospital, Copenhagen University Hospital, Herlev, Denmark; 11 Department of Clinical Biochemistry, Herlev and Gentofte Hospital, Copenhagen University Hospital, Herlev, Denmark; 12 Faculty of Health and Medical Sciences, University of Copenhagen, Copenhagen, Denmark; 13 Centre for Cancer Genetic Epidemiology, Department of Public Health and Primary Care, University of Cambridge, Cambridge, UK; 14 East-Netherlands, Laboratory for Pathology, Hengelo, The Netherlands; 15 Division of Cancer Epidemiology, German Cancer Research Center (DKFZ), Heidelberg, Germany; 16 Cancer Epidemiology Group, University Cancer Center Hamburg (UCCH), University Medical Center Hamburg-Eppendorf, Hamburg, Germany; 17 Department of Medical Epidemiology and Biostatistics, Karolinska Institute, Stockholm, Sweden; 18 Department of Pathology, Leiden University Medical Center, Leiden, The Netherlands; 19 Department of Human Genetics, Leiden University Medical Center, Leiden, The Netherlands; 20 Centre for Cancer Genetic Epidemiology, Department of Oncology, University of Cambridge, Cambridge, UK; 21 Cancer Sciences Academic Unit, Faculty of Medicine, University of Southampton, Southampton, UK; 22 Department of Medicine Division of Hematology and Oncology, University of California at Los Angeles, David Geffen School of Medicine, Los Angeles, CA USA; 23 Department of Gynecology and Obstetrics, Comprehensive Cancer Center ER-EMN, University Hospital Erlangen, Friedrich-Alexander-University Erlangen-Nuremberg, Erlangen, Germany; 24 Usher Institute of Population Health Sciences and Informatics, The University of Edinburgh Medical School, Edinburgh, UK; 25 Cancer Research UK Edinburgh Centre, Edinburgh, UK; 26 Department of Health and Human Services, Division of Cancer Epidemiology and Genetics, National Cancer Institute, National Institutes of Health, Bethesda, MD USA; 27 Department of Breast Surgery, Herlev and Gentofte Hospital, Copenhagen University Hospital, Herlev, Denmark; 28 Division of Genetics and Epidemiology, Institute of Cancer Research, London, UK; 29 Department of Preventive Medicine, Keck School of Medicine, University of Southern California, Los Angeles, CA USA; 30 Department of Oncology, Södersjukhuset, Stockholm, Sweden; 31 Molecular Genetics of Breast Cancer, German Cancer Research Center (DKFZ), Heidelberg, Germany; 32 Centre for Epidemiology and Biostatistics, Melbourne School of Population and Global Health, The University of Melbourne, Melbourne, Victoria Australia; 33 Department of Medical Oncology, Erasmus MC Cancer Institute, Rotterdam, The Netherlands; 34 Department of Genetics and Pathology, Pomeranian Medical University, Szczecin, Poland; 35 Independent Laboratory of Molecular Biology and Genetic Diagnostics, Pomeranian Medical University, Szczecin, Poland; 36 VIB Center for Cancer Biology, VIB, Leuven, Belgium; 37 Laboratory for Translational Genetics, Department of Human Genetics, University of Leuven, Leuven, Belgium; 38 University of Hawaii Cancer Center, Epidemiology Program, Honolulu, HI USA; 39 Department of Molecular Medicine and Surgery, Karolinska Institutet, Stockholm, Sweden; 40 Department of Clinical Genetics, Karolinska University Hospital, Stockholm, Sweden; 41 Unit of Clinical Epidemiology and Trial Organization, Fondazione IRCCS Istituto Nazionale dei Tumori, Milan, Italy; 42 Department of Obstetrics and Gynecology, Helsinki University Hospital, University of Helsinki, Helsinki, Finland; 43 Department of Surgical Oncology, The Netherlands Cancer Institute - Antoni van Leeuwenhoek Hospital, Amsterdam, The Netherlands; 44 Department of Research, Netherlands Comprehensive Cancer Organisation, Utrecht, The Netherlands; 45 Precision Medicine, School of Clinical Sciences at Monash Health, Monash University, Clayton, Victoria Australia; 46 Department of Clinical Pathology, The University of Melbourne, Melbourne, Victoria Australia; 47 Faculty of Medicine, University of Southampton, Southampton, UK; 48 Department of Surgery, Leiden University Medical Center, Leiden, The Netherlands; 49 Department of Pathology, Erasmus MC Cancer Institute, Rotterdam, The Netherlands; 50 Division of Psychosocial Research and Epidemiology, The Netherlands Cancer Institute - Antoni van Leeuwenhoek Hospital, Plesmanlaan 121, 1066 CX Amsterdam, The Netherlands; 51 Leuven Multidisciplinary Breast Center, Department of Oncology, Leuven Cancer Institute, University Hospitals Leuven, Leuven, Belgium; 52 Department of Clinical Science and Education, Södersjukhuset, Karolinska Institutet, Stockholm, Sweden; 53 BOOG, Laboratory for Pathology Dordrecht, Dordrecht, The Netherlands

**Keywords:** Contralateral breast cancer, Risk prediction model, Clinical decision-making, *BRCA* mutation carriers

## Abstract

**Background:**

Breast cancer survivors are at risk for contralateral breast cancer (CBC), with the consequent burden of further treatment and potentially less favorable prognosis. We aimed to develop and validate a CBC risk prediction model and evaluate its applicability for clinical decision-making.

**Methods:**

We included data of 132,756 invasive non-metastatic breast cancer patients from 20 studies with 4682 CBC events and a median follow-up of 8.8 years. We developed a multivariable Fine and Gray prediction model (PredictCBC-1A) including patient, primary tumor, and treatment characteristics and *BRCA1/2* germline mutation status, accounting for the competing risks of death and distant metastasis. We also developed a model without *BRCA1/2* mutation status (PredictCBC-1B) since this information was available for only 6% of patients and is routinely unavailable in the general breast cancer population. Prediction performance was evaluated using calibration and discrimination, calculated by a time-dependent area under the curve (AUC) at 5 and 10 years after diagnosis of primary breast cancer, and an internal-external cross-validation procedure. Decision curve analysis was performed to evaluate the net benefit of the model to quantify clinical utility.

**Results:**

In the multivariable model, *BRCA1/2* germline mutation status, family history, and systemic adjuvant treatment showed the strongest associations with CBC risk. The AUC of PredictCBC-1A was 0.63 (95% prediction interval (PI) at 5 years, 0.52–0.74; at 10 years, 0.53–0.72). Calibration-in-the-large was -0.13 (95% PI: -1.62–1.37), and the calibration slope was 0.90 (95% PI: 0.73–1.08). The AUC of Predict-1B at 10 years was 0.59 (95% PI: 0.52–0.66); calibration was slightly lower. Decision curve analysis for preventive contralateral mastectomy showed potential clinical utility of PredictCBC-1A between thresholds of 4–10% 10-year CBC risk for *BRCA1/2* mutation carriers and non-carriers.

**Conclusions:**

We developed a reasonably calibrated model to predict the risk of CBC in women of European-descent; however, prediction accuracy was moderate. Our model shows potential for improved risk counseling, but decision-making regarding contralateral preventive mastectomy, especially in the general breast cancer population where limited information of the mutation status in *BRCA1/2* is available, remains challenging.

## Introduction

Breast cancer (BC) is a major burden for women’s health [1]. Survival has improved substantially over the past half century due to earlier detection and advanced treatment modalities, for example in the Netherlands, 10-year survival of a first primary BC improved from 40% in 1961–1970 to 79% in 2006–2010 [2]. Consequently, increasing numbers of BC survivors are at risk to develop a new primary tumor in the opposite (contralateral) breast, with subsequent treatment and potentially less favorable prognosis [3]. BC survivors are more likely to develop contralateral breast cancer (CBC) compared to healthy women to develop a first primary BC [4].

Women at elevated CBC risk have been identified to be *BRCA1/2* and *CHEK2* c.1100del mutation carriers and to have a BC family history, particularly a family history of bilateral BC [5–10]. For *BRCA1/2* mutation carriers, in whom CBC risk is high, contralateral preventive mastectomy (CPM) is often offered [11]. However, the average risk of CBC among all first BC survivors is still relatively low, with an incidence of ~ 0.4% per year [12–14]. Despite this, in recent years, CPM frequency has increased among women in whom CBC risk is low [15]. For these reasons, there is an urgent need for improved individualized prediction of CBC risk, both to facilitate shared decision-making of physicians and women regarding treatment and prevention strategies for those at high CBC risk and to avoid unnecessary CPM or surveillance mammography after first primary BC when CBC risk is low.

To our knowledge, only one specific CBC risk prediction model (CBCrisk) has been developed to date. CBCrisk used data on 1921 CBC cases and 5763 matched controls with validation in two independent US studies containing a mix of invasive and in situ BC [16, 17]. Moreover, the level of prediction performance measures such as calibration and discrimination needed for a CBC risk prediction to be clinically useful have not yet been addressed [18].

Our aim was twofold: first, to develop and validate a CBC risk prediction model using a large international series of individual patient data including 132,756 patients with a first primary invasive BC between 1990 and 2013 from multiple studies in Europe, USA, and Australia with 4682 incident CBCs, and second, to evaluate the potential clinical utility of the model to support decision-making.

## Material and methods

### Study population

We used data from five main sources: three studies from the Netherlands, 16 studies from the Breast Cancer Association Consortium (BCAC), and a cohort from the Netherlands Cancer Registry [19–22]. For details regarding data collection and patient inclusion, see Additional file 1: Data and patient selection and Table S1, and Additional file 1: Table S2. We included female patients with invasive non-metastatic first primary BC with no prior history of cancer (except for non-melanoma skin cancer). The studies were either population- or hospital-based series; most women were of European-descent. We only included women diagnosed after 1990 to have a population with diagnostic and treatment procedures likely close to modern practice and at the same time sufficient follow-up to study CBC incidence; in total 132,756 women from 20 studies were included. All studies were approved by the appropriate ethics and scientific review boards. All women provided written informed consent or did not object to secondary use of clinical data in accordance with Dutch legislation and codes of conduct [23, 24].

### Available data and variable selection

Several factors have been shown or suggested to be associated with CBC risk, including age at first BC, family history for BC, *BRCA1/2* and *CHEK2* c.1100del mutations, body mass index (BMI), breast density change, (neo)adjuvant chemotherapy, endocrine therapy, CPM, and characteristics of the first BC such as histology (lobular vs ductal), estrogen receptor (ER) status, lymph node status, tumor size, and TNM stage [5, 9, 12, 25–36]. The choice of factors to include in the analyses was determined by evidence from literature, availability of data in the cohorts, and current availability in clinical practice. We extracted the following information: *BRCA1/2* germline mutation, (first degree) family history of primary BC, and regarding primary BC diagnosis: age, nodal status, size, grade, morphology, ER status, progesterone receptor (PR), human epidermal growth factor receptor 2 (HER2) status, administration of adjuvant and/or neoadjuvant chemotherapy, adjuvant endocrine therapy, adjuvant trastuzumab therapy, radiotherapy. We excluded PR status and TNM stage of the primary BC due to collinearity with ER status and the size of the primary tumor, respectively. In the current clinical practice, only patients with ER-positive tumors receive endocrine therapy and only patients with HER2-positive tumors receive trastuzumab; these co-occurrences were considered in the model by using composite categorical variables. More information is available online about the factors included in the analyses (Additional file 1: Data patient selection and Additional file 2: Figure S1), follow-up per dataset, and study design (Additional file 1: Table S2).

### Statistical analyses

All analyses were performed using SAS (SAS Institute Inc., Cary, NC, USA) and R software [37].

### Primary endpoint, follow-up, and predictors

The primary endpoint in the analyses was in situ or invasive metachronous CBC. Follow-up started 3 months after invasive first primary BC diagnosis, in order to exclude synchronous CBCs, and ended at date of CBC, distant metastasis (but not at loco-regional relapse), CPM, or last date of follow-up (due to death, being lost to follow-up, or end of study), whichever occurred first. The follow-up of 27,155 (20.4%) women from the BCAC studies, recruited more than 3 months after diagnosis of the first primary BC (prevalent cases), started at recruitment (left truncation). Distant metastasis and death due to any cause were considered as competing events. Patients who underwent CPM during the follow-up were censored because the CBC risk was almost zero after a CPM [38]. Missing data were multiply imputed by chained equations (MICE) to avoid loss of information due to case-wise deletion [39, 40]. Details about the imputation model, strategy used, and the complete case analysis are provided in Additional file 1: Multiple Imputation of missing values, complete case analysis, and model diagnostics and baseline recalibration and Additional file 1: Tables S3 and S4.

### Model development and validation

For model development, we used a multivariable Fine and Gray model regression to account for death and distant metastases as competing events [41, 42]. Heterogeneity of baseline risks between studies was taken into account using the study as a stratification term. A stratified model allows the baseline subdistribution hazard to be different across the studies, and parameter estimation is performed by maximization of the partial likelihood per study. A Breslow-type estimator was used to estimate the baseline cumulative subdistribution hazard per study. The assumption of proportional subdistribution hazards was graphically checked using Schoenfield residuals [43]. The resulting subdistributional hazard ratios (sHRs) and corresponding 95% confidence intervals (CI) were pooled from the 10 imputed data sets using Rubin’s rules [44]. We built a nomogram for estimating the 5- and 10-year cumulative incidence of CBC as a graphical representation of the multivariable risk prediction model [45].

The validity of the model was investigated by leave-one-study-out cross-validation, i.e., in each validation step, all studies are used except one in which the validity of the model is evaluated [46, 47]. Since the ABCS study and some studies from BCAC had insufficient CBC events required for reliable validation, we used the geographic area as unit of splitting. We had 20 studies in five main sources: 17 out of 20 studies that were combined in 4 geographic areas. In total, 3 studies and 4 geographic areas were used to assess the prediction performance of the model (see Additional file 1: Leave-one-study-out cross-validation and Additional file 1: Table S5, [47, 48].

The performance of the model was assessed by discrimination ability to differentiate between patients who experienced CBC and those who did not, and by calibration, which measures the agreement between observed and predicted CBC risk. Discrimination was quantified by time-dependent area under the ROC curves (AUCs) based on Inverse Censoring Probability Weighting at 5 and 10 years [49, 50]. In the presence of competing risks, the R package timeROC provides two types of AUC according to a different definition of time-dependent cases and controls. AUCs were calculated considering a patient who developed a CBC as a case and a patient free of any event as a control at 5 and 10 years [50]. Values of AUCs close to 1 indicate good discriminative ability, while values close to 0.5 indicated poor discriminative ability. Calibration was assessed by the calibration-in-the-large and slope statistic [51]. Calibration-in-the-large lower or higher than 0 indicates that prediction is systematically too high or low, respectively. A calibration slope of 1.0 indicates good overall calibration; slopes below (above) 1.0 indicate over (under) estimation of risk by the model.

To allow for heterogeneity among studies, a random-effect meta-analysis was performed to provide summaries of discrimination and calibration performance. The 95% prediction intervals (PI) indicated the likely range for the prediction performances of the model in a new dataset. Further details about the validation process are provided in Additional file 1: Leave-one-study-out cross-validation.

### Clinical utility

The clinical utility of the prediction model was evaluated using decision curve analysis (DCA) [52, 53]. Such a decision may apply to more or less intensive screening and follow-up or to decision of a CPM. The key part of the DCA is the net benefit, which is the number of true-positive classifications (in this example: the benefit of CPM to a patient who would have developed a CBC) minus the number of false-positive classifications (in this example: the harm of unnecessary CPM in a patient who would not have developed a CBC). The false positives are weighted by a factor related to the relative harm of a missed CBC versus an unnecessary CPM. The weighting is derived from the threshold probability to develop a CBC using a defined landmark time point (e.g., CBC risk at 5 or 10 years) [54]. For example, a threshold of 10% implies that CPM in 10 patients, of whom one would develop CBC if untreated, is acceptable (thus performing 9 unnecessary CPMs). The net benefit of a prediction model is traditionally compared with the strategies of treat all or treat none. Since the use of CPM is generally only suggested among *BRCA1/2* mutation carriers, for a more realistic illustration, the decision curve analysis was reported among *BRCA1/2* mutation carriers and non-carriers [55]. See Additional file 1: Clinical utility for details.

## Results

A total of 132,756 invasive primary BC women diagnosed between 1990 and 2013, with 4682 CBC events, from 20 studies, were used to derive the model for CBC risk (Additional file 1: Table S2). Median follow-up time was 8.8 years, and CBC cumulative incidences at 5 and 10 years were 2.1% and 4.1%, respectively. Details of the studies and patient, tumor, and treatment characteristics are provided in Additional file 1: Table S6. The multivariable model with estimates for all included factors is shown in Table 1 and Additional file 3. *BRCA1/2* germline mutation status, family history, and systemic adjuvant treatment showed the strongest associations with CBC risk.

**Table 1:** Multivariable subdistribution hazard model for contralateral breast cancer risk

Factor (category) at primary breast cancer	Multivariable analysis	
	sHR	95% CI
Age, *years*	0.68*	0.62–0.74*
Family history (yes versus no)	1.35	1.27–1.45
*BRCA* mutation		
*BRCA1* versus non-carrier	3.68	3.34–4.07
*BRCA2* versus non-carrier	2.56	2.36–2.78
Nodal status (positive versus negative)	0.87	0.80–0.93
Tumor size, *cm*		
2.5 versus ≤ 2	0.95	0.89–1.02
> 5 versus ≤ 2	1.14	0.99–1.31
Morphology (lobular including mixed versus ductal including others)	1.23	1.14–1.34
Grade		
Moderately differentiated versus well differentiated	0.89	0.82–0.96
Poorly differentiated versus well differentiated	0.75	0.70–0.82
Chemotherapy (yes versus no)	0.77	0.70–0.84
Radiotherapy to the breast (yes versus no)	1.01	0.95–1.08
ER (positive or negative)/endocrine therapy (yes or no)		
Negative/no versus positive/yes	1.43	1.30–1.57
Positive/no versus positive/yes	1.75	1.61–1.90
HER2 (positive or negative)/trastuzumab therapy (yes or no)		
Negative/no versus positive/yes	1.08	0.93–1.27
Positive/no versus positive/yes	0.99	0.83–1.18

*sHR* subdistributional hazard ratio, *CI* confidence interval, *ER* estrogen receptor, *HER2* human epidermal growth factor receptor 2. *Age was parameterized as a linear spline with one interior knot at 50 years. For representation purposes, we here provide the sHR for the 75th versus the 25th percentile. For more details about age parameterization, see also Additional file 3: Supplementary Methods

**Fig. 1:**
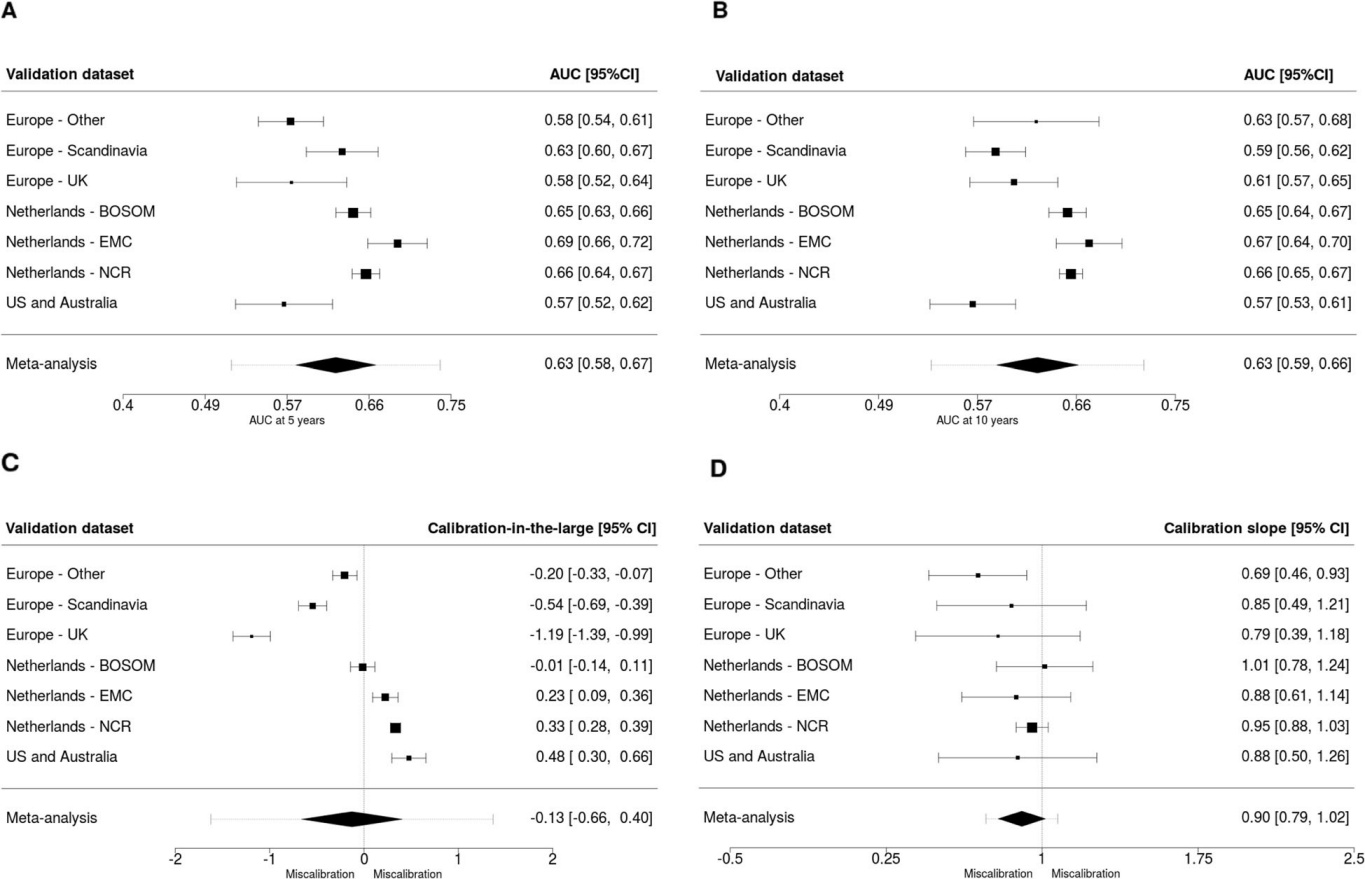
Analysis of predictive performance in leave-one-study-out cross-validation. **a**, **b** The discrimination assessed by a time-dependent AUC at 5 and 10 years, respectively. **c** The calibration accuracy measured with calibration-in-the-large. **d** The calibration accuracy measured with calibration slope. The black squares indicate the estimated accuracy of a model built using all remaining studies or geographic areas. The black horizontal lines indicate the corresponding 95% confidence intervals of the estimated accuracy (interval whiskers). The black diamonds indicate the mean with the corresponding 95% confidence intervals of the predictive accuracy, and the dashed horizontal lines indicate the corresponding 95% prediction intervals

The prediction performance of the main model (PredictCBC, version 1A) based on the leave-one-study-out cross-validation method is shown in Fig. 1. The AUC at 5 years was 0.63 (95% confidence interval (CI): 0.58–0.67; 95% prediction interval (PI): 0.52–0.74)); the AUC at 10 years was also 0.63 (95% CI: 0.59–0.66; 95% PI: 0.53–0.72). Calibrations showed some indications of overestimation of risk. The calibration-in-the-large was − 0.13 (95% CI: -0.66–0.40; 95% PI: -1.62–1.37). The calibration slope was 0.90 (95% CI: 0.79–1.02; 95% PI: 0.73–1.08) in the cross-validation. Calibration plots are provided in Additional file 2: Figure S2 and S3.

**Fig. 2:**
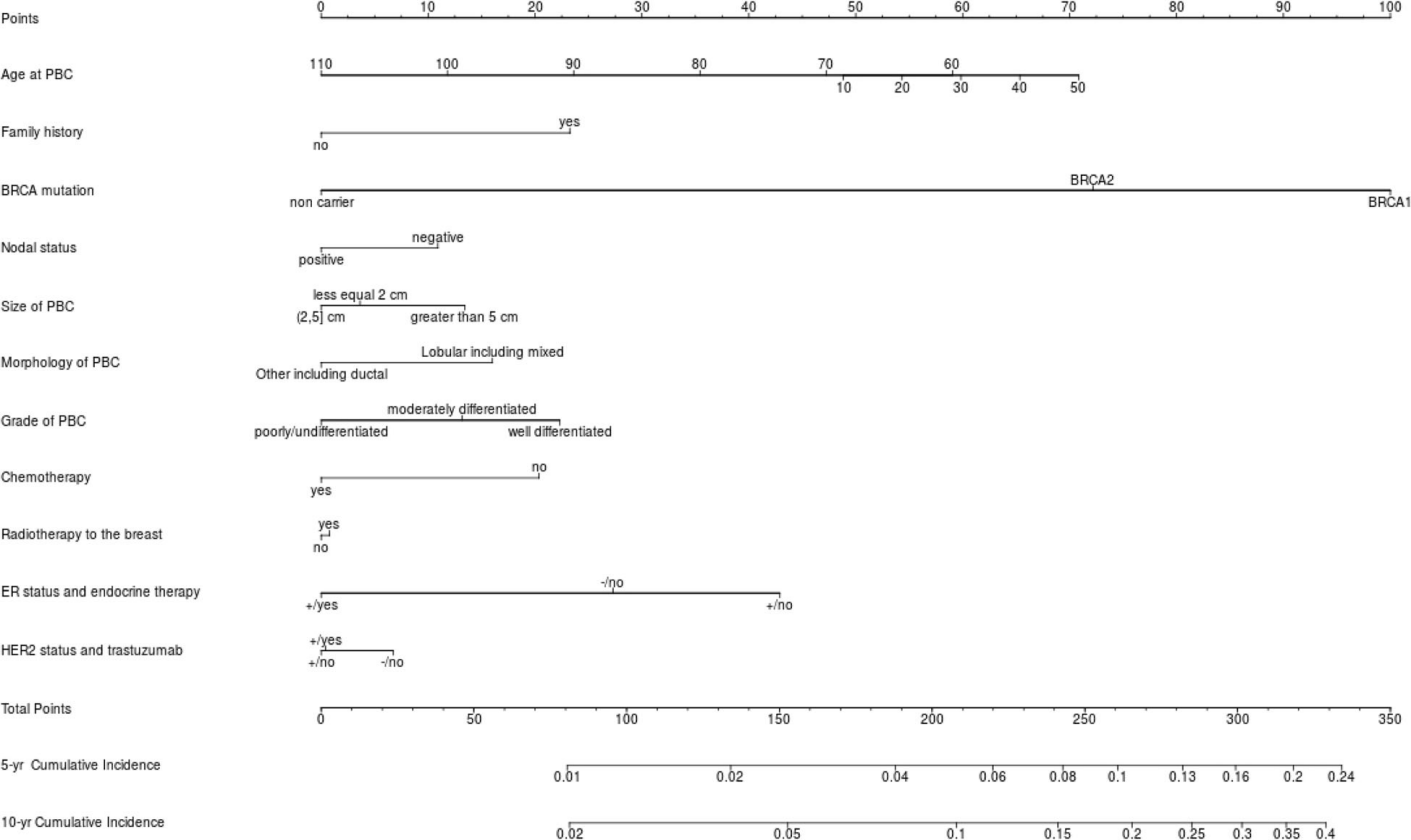
Nomogram for the prediction of 5- and 10-year contralateral breast cancer cumulative incidence. The 5- and 10-year contralateral breast cancer cumulative incidence is calculated by taking the sum of the risk points, according to patient, first primary breast cancer tumor, and treatment characteristics. For each factor, the number of associated risk points can be determined by drawing a vertical line straight up from the factor’s corresponding value to the axis with risk points (0–100). The total points axis (0–350) is the sum of the factor’s corresponding values determined by every individual patient’s characteristics. Draw a line straight down from the total points axis to find the 5- and 10-year cumulative incidence. PBC primary breast cancer, ER estrogen receptor status, HER2 human epidermal growth factor receptor 2, yr year

The nomogram representing a graphical tool for estimating the CBC cumulative incidence at 5 and 10 years based on our model and the estimated baseline of the Dutch Cancer Registry is shown in Fig. 2. In the nomogram, the categories of each factor are assigned a score using the topmost “Points” scale, then all scores are summed up to obtain the “Total points”, which relate to the cumulative incidence of CBC. The formulae of the models (PredictCBC-1A and 1B) providing the predicted cumulative incidence are given in Additional file 1: Formula to estimate the CBC risk and formula to estimate CBC risk in patients not tested for *BRCA*.

**Fig. 3:**
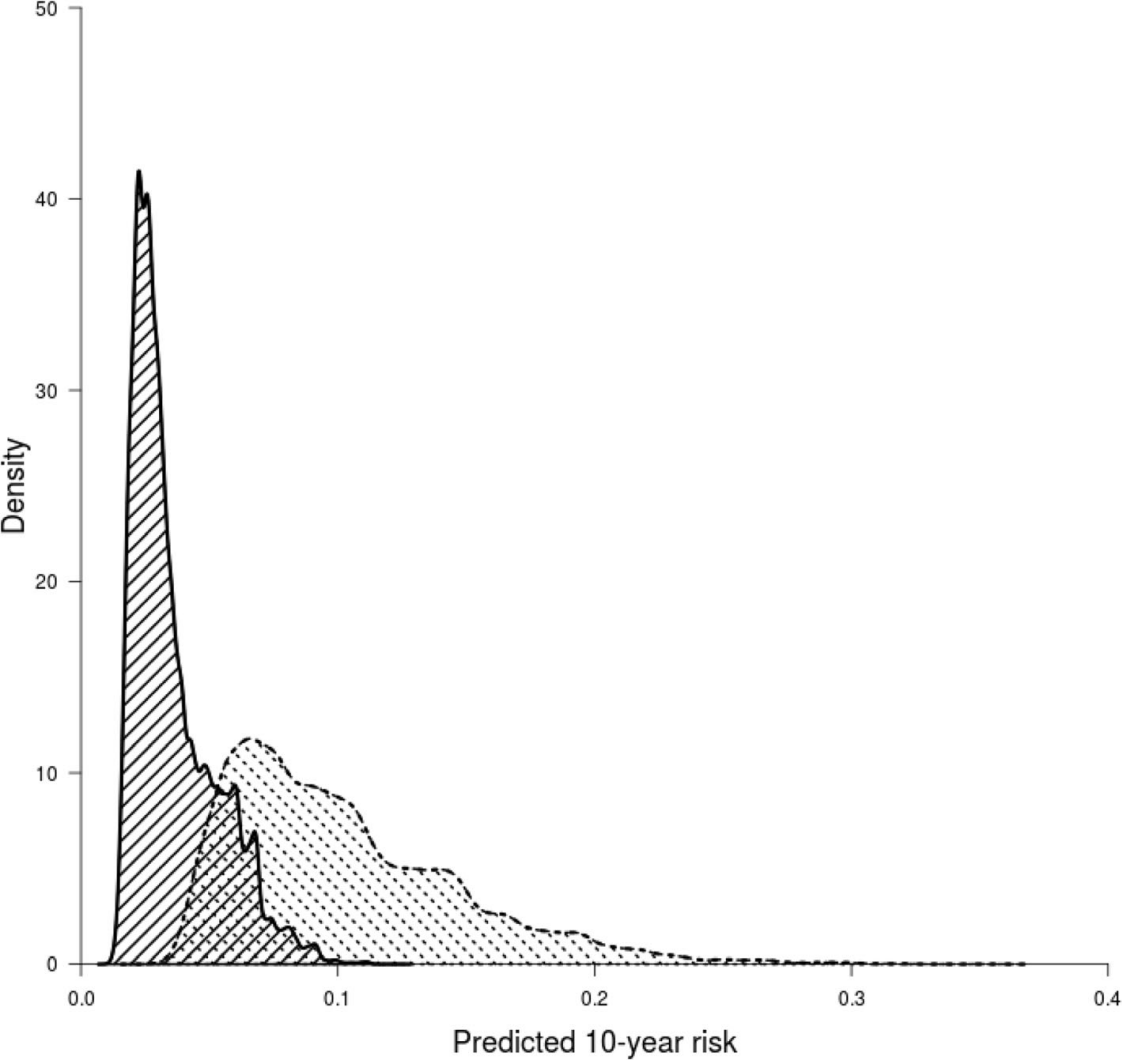
Density distribution of 10-year predicted contralateral breast cancer absolute risk within non-carriers (area with black solid lines) and *BRCA1/2* mutation carriers (area with black dashed lines)

**Fig. 4:**
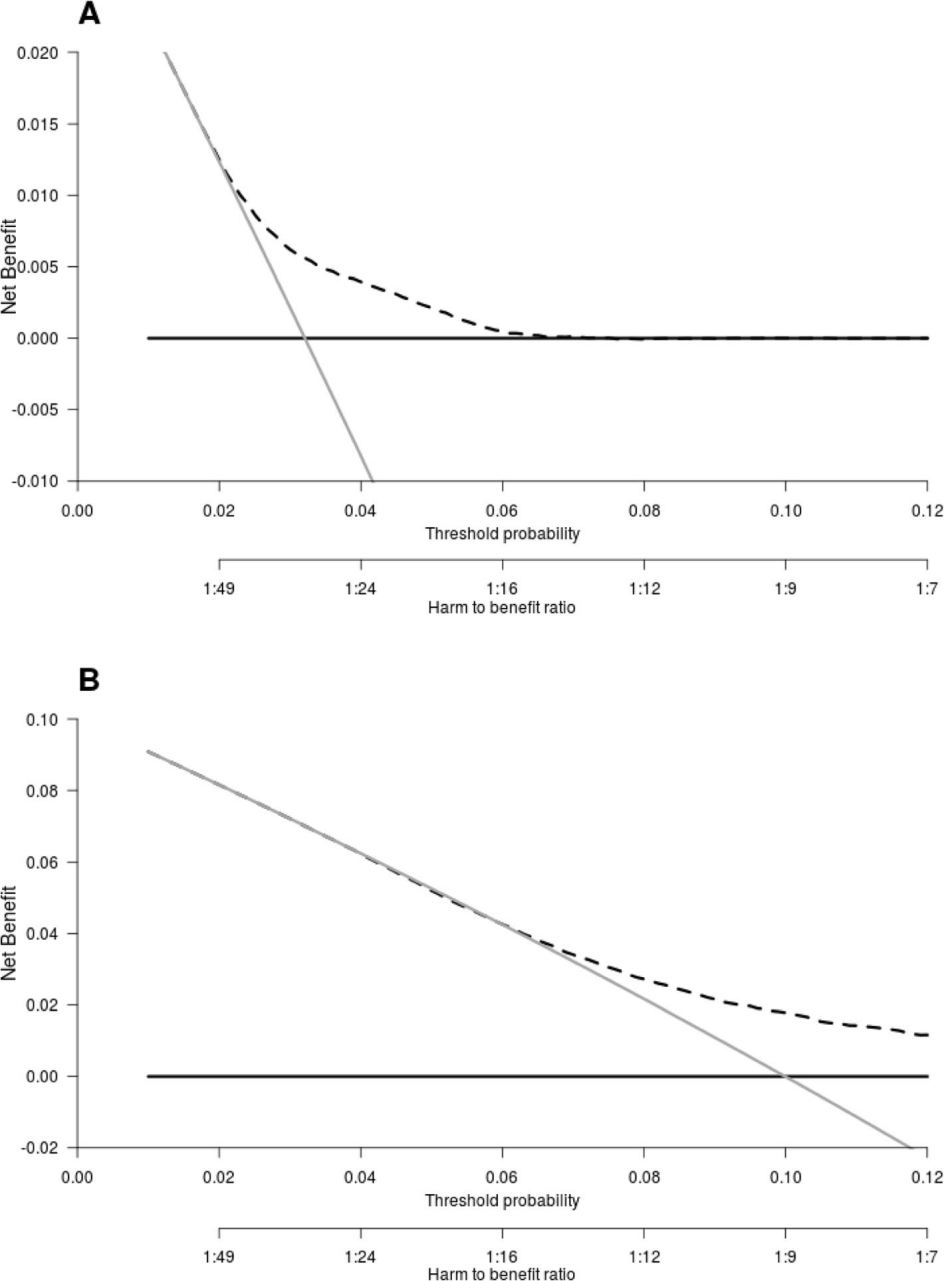
Decision curve analysis at 10 years for the contralateral breast cancer risk model including *BRCA* mutation information. **a** The decision curve to determine the net benefit of the estimated 10-year predicted contralateral breast cancer (CBC) cumulative incidence for patients without a *BRCA1/2* gene mutation using the prediction model (dotted black line) compared to not treating any patients with contralateral preventive mastectomy (CPM) (black solid line). **b** The decision curve to determine the net benefit of the estimated 10-year predicted CBC cumulative incidence for *BRCA1/2* mutation carriers using the prediction model (dotted black line) versus treating (or at least counseling) all patients (gray solid line). The *y*-axis measures net benefit, which is calculated by summing the benefits (true positives, i.e., patients with a CBC who needed a CPM) and subtracting the harms (false positives, i.e., patients with CPM who do not need it). The latter are weighted by a factor related to the relative harm of a non-prevented CBC versus an unnecessary CPM. The factor is derived from the threshold probability to develop a CBC at 10 years at which a patient would opt for CPM (e.g., 10%). The *x*-axis represents the threshold probability. Using a threshold probability of 10% implicitly means that CPM in 10 patients of whom one would develop a CBC if untreated is acceptable (9 unnecessary CPMs, harm to benefit ratio 1:9)

The DCAs for preventive contralateral mastectomy showed the potential clinical utility of PredictCBC-1A between thresholds of 4–10% 10-year CBC risk for *BRCA1/2* mutation carriers and non-carriers (Table 2 and Additional file 3). For example, if we find it acceptable that one in 10 patients for whom a CPM is recommended develops a CBC, a risk threshold of 10% may be used to define high and low risk *BRCA1/2* mutation carriers based on the absolute 10-year CBC risk prediction estimated by the model. Compared with a strategy recommending CPM to all carriers of a mutation in *BRCA1*/2, this strategy avoids 161 CPMs per 1000 patients. In contrast, almost no non *BRCA1/2* mutation carriers reach the 10% threshold (the general BC population, Fig. 3). The decision curves provide a comprehensive overview of the net benefit for a range of harm-benefit thresholds at 10-year CBC risk (Fig. 4).

**Table 2:** Clinical utility of the 10-year contralateral breast cancer risk prediction model. At the same probability threshold, the net benefit is exemplified in *BRCA1/2* mutation carriers (for avoiding unnecessary CPM) and non-carriers (performing necessary CPM)

Probability threshold, *p*_t_ (%)	Unnecessary CPMs needed to prevent one CBC*	*BRCA1/2* mutation carriers		Non-carriers	
		Net benefit versus treat all patients with CPM (per 1000)	Avoided unnecessary CPMs per 1000 patients	Net benefit versus treat none (per 1000)	Performed necessary CPMs per 1000 patients
4	24.0	0.0	0.0	3.9	93.6
5	19.0	0.0	0.0	2.1	39.9
6	15.7	0.1	1.6	0.5	7.8
7	13.3	1.9	25.2	0.1	1.3
8	11.5	5.5	63.3	0.0	0.0
9	10.1	10.7	108.2	0.0	0.0
10	9.0	17.9	161.1	0.0	0.0

*CPM* contralateral preventive mastectomy, *CBC* contralateral breast cancer. *The number of unnecessary contralateral mastectomies needed to prevent a CBC is calculated by (1 − *p*_t_)/*p*_t_. See also Additional file 3: Methods

Decision curves for CBC risk at 5 year and the corresponding clinical utility are provided in Additional file 2: Figure S4 and Additional file 1: Table S7, respectively.

We also derived a risk prediction model (PredictCBC, version 1B) omitting *BRCA* status to provide CBC risk estimates for first BC patients not tested for *BRCA1/2* mutations. This model has slightly lower prediction performance; AUC at 5 and 10 years was both 0.59 (at 5 years: 95% CI: 0.54–0.63, 95% PI: 0.46–0.71; at 10 years: 95% CI: 0.56–0.62, 95% PI: 0.52–0.66), calibration-in-the-large was − 0.17 (95% CI: -0.72–0.38; 95% PI: -1.70–1.36), and calibration slope was 0.81 (95% CI 0.63–0.99; 95% PI: 0.50–1.12) (Additional file 1: Results of the prediction model without *BRCA* mutation). Details of development, validation, and clinical utility are provided in Additional file 1: Tables S8–S10 and Figure S5–S10.

In a sensitivity analysis (see Additional file 1: Assessment of limited information of CPM), we studied the impact of CPM on our results using two studies, in which CPM information was (almost) completely available. The lack of CPM information on cumulative incidence estimation hardly affected the results of our analyses (Additional file 2: Figure S11).

## Discussion

Using established risk factors for CBC which are currently available in clinical practice, we developed PredictCBC, which can be used to calculate 5- and 10-year absolute CBC risk. The risk prediction model includes carriership of *BRCA1/2* mutations, an important determinant of CBC risk in the decision-making process [6].

The calibration of the model was reasonable and discrimination moderate within the range of other tools commonly used for routing counseling and decision-making in clinical oncology for primary BC risk [56–59]. As expected, the prediction accuracy was lower when we omitted the *BRCA* mutation carrier status although the prevalence of *BRCA* mutations among BC patients is quite low (2–4%) [60, 61].

In the breast cancer population, CBC is a relatively uncommon event (~ 0.4% per year) and difficult to predict. Therefore, physicians should carefully consider which patients should consider CPM using a prediction model [62]. The current clinical recommendations of CPM are essentially based on the presence of a mutation in the *BRCA1/2* genes. Based on the risk distribution defined by the current model (Fig. 3), this is a reasonable approach: essentially no non-carrier women reach a 10% risk 10-year threshold. However, more than 50% of carriers do not reach this threshold either, suggesting that a significant proportion of *BRCA1/2* carriers might be spared CPM. Contralateral surveillance mammography may also be avoided although detection and knowledge of recurrences may be necessary for better defined individualized follow-up and patient-tailored treatment strategies [63, 64].

CBC risk patterns and factors were identified previously in a large population-based study with 10,944 CBC of 212,630 patients from the Surveillance, Epidemiology and End Results (SEER) database diagnosed from 1990 to 2013 [65]. However, SEER does not include details of endocrine treatment and chemotherapy, therapies administrated to reduce recurrences and CBCs [13, 66]. Furthermore, in this study, the model was not validated or evaluated based on prediction accuracy, nor was a tool provided. Another study provided general guidelines for CPM by calculating the lifetime risk of CBC based on a published systematic review of age at first BC, *BRCA1/2* gene mutation, family history of BC, ER status, ductal carcinoma in situ, and oophorectomy [34, 67]. However, the authors specified that the calculation of the CBC lifetime risk should be considered only as a guide for helping clinicians to stratify patients into risk categories rather than a precise tool for the objective assessment of the risk.

Only one other prediction model (CBCrisk) has been developed and validated using data of 1921 CBC cases and 5763 matched controls [16]. External validation of CBCrisk of two independent datasets using 5185 and 6035 patients with 111 and 117 CBC assessed a discrimination between 0.61 and 0.65 [17]. The discrimination of our PredictCBC model at 5 and 10 years was similar; however, the geographic diversity of the studies gave a more complete overview of external validity [47]. Moreover, we showed the net benefit of our model using decision curve analysis since standard performance metrics of discrimination, calibration, sensitivity, and specificity alone are insufficient to assess the clinical utility [18, 53].

Some limitations of our study must be recognized. First, reporting of CBC was not entirely complete in all studies and information about CPM was limited in most datasets, which may have underestimated the cumulative incidence, although the overall 10-year cumulative incidence of 4.1% is in line with other data [5, 34]. Second, some women included in the Dutch studies (providing specific information on family history, *BRCA* mutation, or CPM) were also present in our selection of the Netherlands Cancer Registry population. Privacy and coding issues prevented linkage at the individual patient level, but based on the hospitals from which the studies recruited, and the age and period criteria used, we calculated a maximum potential overlap of 3.4%. Third, in the US and Australian datasets, the prediction performance was uncertain due to the limited sample size and missing values. Moreover, some important predictors such as family history and especially *BRCA* mutation status were only available in a subset of the women (from familial- and unselected hospital-based studies) and patients with data on *BRCA* mutation status might have been insufficiently represented for tested populations and further development and validation of PredictCBC-1A will be necessary. However, although *BRCA1/2* mutation information was unavailable in 94% of our data, the approach of the imputation led to consistently good performing models [68–70]. The remaining factors were quite complete: ~ 79% of patients had at most one missing factor, which provided good imputation diagnostic performances. Since most BC patients are not currently tested in the clinical practice for *BRCA1/2* mutations, we assessed the clinical utility of PredictCBC version 1B to provide individualized CBC risk estimates for first BC patients not tested for *BRCA1/2* germline mutations [60, 71]. Our PredictCBC version 1B model provides less precise estimates, but may be useful in providing general CBC risk estimates, which could steer women away from CPM or trigger *BRCA* testing.

Last but not the least, adequate presentation of the risk estimates from the PredictCBC-1A and PredictCBC-1B is crucial for effective communication about CBC risk during doctor-patient consultations [72, 73]. A nomogram is an important component to communicate the risk of modern medical decision-making, although it may be difficult to use and might potentially make it more difficult to interpret the risks for laymen [74] An online tool is being implemented, and a pilot study will be conducted among patients and clinicians to assess how the risk estimates from PredictCBC-1A and 1B can best be visualized to facilitate communication with patients. Other factors, which were not available in our study, predict breast cancer risk and their inclusion may further improve the discrimination and clinical utility of our CBC risk model: these factors include *CHEK2* c.1100del mutation carriers, polygenic risk scores based on common genetic variants, breast density, and reproductive and lifestyle factors such as BMI and age at menarche [75]. Additional data with complete information of *BRCA1/2* mutation should be also considered in the model upgrade to reduce uncertainty of CBC risk estimates. External validation in other studies, including patients of other ethnicities, will also be important. In the meantime, our model provides a reliable basis for CBC risk counseling.

## Conclusions

In conclusion, we have developed and cross-validated risk prediction models for CBC (PredictCBC) based on different European-descent population and hospital-based studies. The model is reasonably calibrated and prediction accuracy is moderate. The clinical utility assessment of PredictCBC showed potential for improved risk counseling, although the decision regarding CPM in the general breast cancer population remains challenging. Similar results have been found for PredictCBC version 1B, a CBC risk prediction model that calculates individualized CBC risk for first BC patients not tested for *BRCA1/2* germline mutation.

## Supplementary information

**Additional file 1 Table S1.** Data source flowchart. **Table S2.** Description of the studies included in the analyses. **Table S3.** Patients and first primary breast cancer characteristics used in the contralateral breast cancer risk prediction model in the complete case and all case analyses. **Table S4.** Results of multivariable subdistributional hazard model using the complete case dataset. **Table S5.** List of BCAC studies (including ABCS source) with the corresponding country and geographic area. **Table S6.** Main patient and disease characteristics. **Table S7.** Clinical utility of the 5-year contralateral breast cancer risk prediction model. **Table S8.** Results of multivariable subdistributional hazard model for breast cancer patients without *BRCA* mutations. **Table S9.** Clinical utility of the 5-year contralateral breast cancer risk prediction model in non-*BRCA* tested patients. **Table S10.** Clinical utility of the 10-year contralateral breast cancer risk prediction model in non-*BRCA* tested patients.

**Additional file 2 Figure S1.** Graphical assessment of non-linear relationship of age with contralateral breast cancer risk. **Figure S2.** Visual assessment of calibration through calibration plots in the internal-external cross-validation at 5 years for the contralateral breast cancer risk model with *BRCA* mutation information. **Figure S3.** Visual assessment of calibration through calibration plots in the internal-external cross-validation at 10 years for the contralateral breast cancer risk model with *BRCA* mutation information. **Figure S4.** Decision curve analysis at 5 years for the contralateral breast cancer risk model including *BRCA1/2* mutation information. **Figure S5.** Results of the leave-one-study-out cross-validation for the contralateral breast cancer risk model at 5 and 10 years without *BRCA* mutation information. **Figure S6.** Visual assessment of calibration through calibration plots in the internal-external cross-validation at 5 years for the contralateral breast cancer risk model without *BRCA* mutation information. **Figure S7.** Visual assessment of calibration through calibration plots in the internal-external cross-validation at 10 years for the contralateral breast cancer risk model without *BRCA* mutation information. **Figure S8.** Density distribution of 10-year predicted absolute risk in patients with no family history and patients with a family history. **Figure S9.** Decision curve analysis at 5 years for the contralateral breast cancer risk model without *BRCA* mutation information. **Figure S10.** Decision curve analysis at 10 years for the contralateral breast cancer risk model without *BRCA* mutation information. **Figure S11.** Assessment of inclusion of information of contralateral preventive mastectomy (CPM).

**Additional file 3.** Supplementary methods.

## Data Availability

All data relevant to this report are included in this published article and its supplementary information files. The datasets analyzed during the current study are not publicly available due to protection of participant privacy and confidentiality, and ownership of the contributing institutions, but may be made available in an anonymized form via the corresponding author on reasonable request and after approval of the involved institutions.

## Abbreviations

AUC: Area under the ROC curve
BC: Breast cancer
BCAC: Breast Cancer Association Consortium
BMI: Body mass index
CBC: Contralateral breast cancer
CI: Confidence interval
CPM: Contralateral preventive mastectomy
DCA: Decision curve analysis
ER: Estrogen receptor
HER2: Human epidermal growth receptor 2
MICE: Multiple imputation by chained equations
PI: Prediction interval
PR: Progesterone receptor
SEER: Surveillance, Epidemiology and End Results
TNM: TNM Classification of Malignant Tumors
