# Supplementary material for: Prediction and clinical utility of a contralateral breast cancer risk model
Source: Breast Cancer Res. 2019 Dec 17;21:144. doi: 10.1186/s13058-019-1221-1 (PMC6918633; doi:10.1186/s13058-019-1221-1)
Supplement: Supplementary file 3 — Additional file 3. Supplementary methods. [file 13058_2019_1221_MOESM3_ESM.docx]

**Supplementary Materials**

**Prediction and clinical utility of a contralateral breast cancer risk model**

**Authors:** Daniele Giardiello^1, 2^, Ewout W. Steyerberg^2, 3^, Michael Hauptmann^4, 5^, Muriel A. Adank^6^, Delal Akdeniz^7^, Carl Blomqvist^8, 9^, Stig E. Bojesen^10-12^, Manjeet K. Bolla^13^, Mariël Brinkhuis^14^, Jenny Chang-Claude^15, 16^, Kamila Czene^17^, Peter Devilee^18, 19^, Alison M. Dunning^20^, Douglas F. Easton^13, 20^, Diana M. Eccles^21^, Peter A. Fasching^22, 23^, Jonine Figueroa^24-26^, Henrik Flyger^27^, Montserrat García-Closas^26, 28^, Lothar Haeberl^e23^, Christopher A. Haiman^29^, Per Hall^17, 30^, Ute Hamann^31^, John L. Hopper^32^, Agnes Jager^33^, Anna Jakubowska^34, 35^, Audrey Jung^15^, Renske Keeman^1^, Iris Kramer^1^, Diether Lambrechts^36, 37^, Loic Le Marchand^38^, Annika Lindblom^39, 40^, Jan Lubiński^34^, Mehdi Manoochehri^31^, Luigi Mariani^41^, Heli Nevanlinna^42^, Hester S.A. Oldenburg^43^, Saskia Pelders^7^, Paul D.P. Pharoah^13, 20^, Mitul Shah^20^, Sabine Siesling^44^, Vincent T.H.B.M. Smit^18^, Melissa C. Southey^45, 46^, William J. Tapper^47^, Rob A.E.M. Tollenaar^48^, Alexandra J. van den Broek^1^, Carolien H.M. van Deurzen^49^, Flora E. van Leeuwen^50^, Chantal van Ongeval^51^, Laura J. Van't Veer^1^, Qin Wang^13^, Camilla Wendt^52^, Pieter J. Westenend^53^, Maartje J. Hooning^7^, Marjanka K. Schmidt^1, 50^

^1^ The Netherlands Cancer Institute - Antoni van Leeuwenhoek Hospital, Division of Molecular Pathology, Amsterdam, The Netherlands.

^2^ Leiden University Medical Center, Department of Biomedical Data Sciences, Leiden, The Netherlands.

^3^ Erasmus MC Cancer Institute, Department of Public Health, Rotterdam, The Netherlands.

^4^ Brandenburg Medical School, Institute of Biometry and Registry Research, Neuruppin, Germany.

^5^ The Netherlands Cancer Institute - Antoni van Leeuwenhoek Hospital, Department of Epidemiology and Biostatistics, Amsterdam, The Netherlands.

^6^ The Netherlands Cancer Institute - Antoni van Leeuwenhoek hospital, Family Cancer Clinic, Amsterdam, The Netherlands.

^7^ Erasmus MC Cancer Institute, Department of Medical Oncology, Family Cancer Clinic, Rotterdam, The Netherlands.

^8^ University of Helsinki, Department of Oncology, Helsinki University Hospital, Helsinki, Finland.

^9^ Örebro University Hospital, Department of Oncology, Örebro, Sweden.

^10^ Copenhagen University Hospital, Copenhagen General Population Study, Herlev and Gentofte Hospital, Herlev, Denmark.

^11^ Copenhagen University Hospital, Department of Clinical Biochemistry, Herlev and Gentofte Hospital, Herlev, Denmark.

^12^ University of Copenhagen, Faculty of Health and Medical Sciences, Copenhagen, Denmark.

^13^ University of Cambridge, Centre for Cancer Genetic Epidemiology, Department of Public Health and Primary Care, Cambridge, UK.

^14^ East-Netherlands, Laboratory for Pathology, Hengelo, The Netherlands.

^15^ German Cancer Research Center (DKFZ), Division of Cancer Epidemiology, Heidelberg, Germany.

^16^ University Medical Center Hamburg-Eppendorf, Cancer Epidemiology Group, University Cancer Center Hamburg (UCCH), Hamburg, Germany.

^17^ Karolinska Institutet, Department of Medical Epidemiology and Biostatistics, Stockholm, Sweden.

^18^ Leiden University Medical Center, Department of Pathology, Leiden, The Netherlands.

^19^ Leiden University Medical Center, Department of Human Genetics, Leiden, The Netherlands.

^20^ University of Cambridge, Centre for Cancer Genetic Epidemiology, Department of Oncology, Cambridge, UK.

^21^ University of Southampton, Cancer Sciences Academic Unit, Faculty of Medicine, Southampton, UK.

^22^ University of California at Los Angeles, David Geffen School of Medicine, Department of Medicine Division of Hematology and Oncology, Los Angeles, CA, USA.

^23^ University Hospital Erlangen, Friedrich-Alexander-University Erlangen-Nuremberg, Department of Gynecology and Obstetrics, Comprehensive Cancer Center ER-EMN, Erlangen, Germany.

^24^ The University of Edinburgh Medical School, Usher Institute of Population Health Sciences and Informatics, Edinburgh, UK.

^25^ Cancer Research UK Edinburgh Centre, Edinburgh, UK.

^26^ National Cancer Institute, National Institutes of Health, Department of Health and Human Services, Division of Cancer Epidemiology and Genetics, Bethesda, MD, USA.

^27^ Copenhagen University Hospital, Department of Breast Surgery, Herlev and Gentofte Hospital, Herlev, Denmark.

^28^ Institute of Cancer Research, Division of Genetics and Epidemiology, London, UK.

^29^ University of Southern California, Department of Preventive Medicine, Keck School of Medicine, Los Angeles, CA, USA.

^30^ Södersjukhuset, Department of Oncology, Stockholm, Sweden.

^31^ German Cancer Research Center (DKFZ), Molecular Genetics of Breast Cancer, Heidelberg, Germany.

^32^ The University of Melbourne, Centre for Epidemiology and Biostatistics, Melbourne School of Population and Global Health, Melbourne, Victoria, Australia.

^33^ Erasmus MC Cancer Institute, Department of Medical Oncology, Rotterdam, The Netherlands.

^34^ Pomeranian Medical University, Department of Genetics and Pathology, Szczecin, Poland.

^35^ Pomeranian Medical University, Independent Laboratory of Molecular Biology and Genetic Diagnostics, Szczecin, Poland.

^36^ VIB, VIB Center for Cancer Biology, Leuven, Belgium.

^37^ University of Leuven, Laboratory for Translational Genetics, Department of Human Genetics, Leuven, Belgium.

^38^ University of Hawaii Cancer Center, Epidemiology Program, Honolulu, HI, USA.

^39^ Karolinska Institutet, Department of Molecular Medicine and Surgery, Stockholm, Sweden.

^40^ Karolinska University Hospital, Department of Clinical Genetics, Stockholm, Sweden.

^41^ Fondazione IRCCS Istituto Nazionale dei Tumori, Unit of Clinical Epidemiology and Trial Organization, Milan, Italy.

^42^ University of Helsinki, Department of Obstetrics and Gynecology, Helsinki University Hospital, Helsinki, Finland.

^43^ The Netherlands Cancer Institute - Antoni van Leeuwenhoek Hospital, Department of Surgical Oncology, Amsterdam, The Netherlands.

^44^ Netherlands Comprehensive Cancer Organisation, Department of Research, Utrecht, The Netherlands.

^45^ Monash University, Precision Medicine, School of Clinical Sciences at Monash Health, Clayton, Victoria, Australia.

^46^ The University of Melbourne, Department of Clinical Pathology, Melbourne, Victoria, Australia.

^47^ University of Southampton, Faculty of Medicine, Southampton, UK.

^48^ Leiden University Medical Center, Department of Surgery, Leiden, The Netherlands.

^49^ Erasmus MC Cancer Institute, Department of Pathology, Rotterdam, The Netherlands.

^50^ The Netherlands Cancer Institute - Antoni van Leeuwenhoek Hospital, Division of Psychosocial Research and Epidemiology, Amsterdam, The Netherlands.

^51^ Leuven Cancer Institute, University Hospitals Leuven, Leuven Multidisciplinary Breast Center, Department of Oncology, Leuven, Belgium.

^52^ Karolinska Institutet, Department of Clinical Science and Education, Södersjukhuset, Stockholm, Sweden.

^53^ Laboratory for Pathology, Dordrecht, The Netherlands.

Contents

[Supplementary Methods 5](#_Toc20139299)

[*1. Data and patient selection* 5](#_Toc20139300)

[*2. Multiple imputation of missing values* 6](#_Toc20139301)

[*3. Complete case analysis* 8](#_Toc20139302)

[*4. Model diagnostics and baseline recalibration* 8](#_Toc20139303)

[*5. Leave-one-study-out cross-validation* 8](#_Toc20139304)

[*6. Clinical utility* 9](#_Toc20139305)

[*7. Formula to estimate the contralateral breast cancer risk* 11](#_Toc20139306)

[*8. Results of the prediction model without BRCA mutation* 12](#_Toc20139307)

[*9. Formula to estimate the contralateral breast cancer risk in patients not tested for BRCA* 13](#_Toc20139308)

[*10. Assessment of limited information of contralateral preventive mastectomy (CPM)* 14](#_Toc20139309)

[*References* 15](#_Toc20139310)

[Supplementary Tables 16](#_Toc20139311)

[Supplementary Figures 24](#_Toc20139312)

# Supplementary Methods

## *1. Data and patient selection*

For this study we used data from five main sources available from national and international collaborations including nationwide registry data, as well as studies with more detailed information on relevant prediction factors[1-5]. Briefly, the five main sources were: (1) The Breast Cancer Association Consortium (BCAC), which is an international consortium of 102 studies comprising 182,898 patients (data version: January 2017) with a primary breast cancer (BC) diagnosed between 1939 and 2016[1]; (2) The Amsterdam Breast Cancer Study (ABCS) containing 2,390 patients diagnosed with a first BC at the Netherlands Cancer Institute – Antoni van Leeuwenhoek (NKI-AVL) hospital in Amsterdam from 2003 to 2013[2]; (3) The Breast Cancer Outcome Study of Mutation carriers (BOSOM), which is a Dutch consecutive series of 7,106 patients with invasive BC treated for their primary BC in ten centers throughout the Netherlands between 1970 and 2003; in this study 94% of patients were genotyped for *BRCA1/2* germline mutations[3]; (4) The Erasmus Medical Center (EMC) study containing patients diagnosed with BC between 1989 and 2013 who were treated at the EMC in Rotterdam; for this study, complete follow-up was obtained for 3,483 patients that had been diagnosed between 2000 and 2009; (5) The Netherlands Cancer Registry (NCR), which is an ongoing nationwide population-based data registry of all newly diagnosed cancer patients in the Netherlands since 1989[4]. We included patients diagnosed between 2003 and 2010, a period for which sufficient follow-up and receptor status information were provided[4, 5]. The eligibility criteria applied in each data source is reported in **Table S1**. Data were harmonized by recoding each of the main datasets by the responsible data managers according to a standardized data dictionary. We performed checks for data consistency and validity centrally.

We extracted the following information: *BRCA1/2* germline mutation, family history (first degree) of primary BC, and regarding primary BC diagnosis: age, nodal status, size, grade, morphology, estrogen-receptor (ER) status, progesterone-receptor (PR), human epidermal growth factor receptor 2 (HER2) status, administration of adjuvant or neoadjuvant chemotherapy, adjuvant endocrine therapy, adjuvant trastuzumab therapy, radiotherapy. We excluded PR status and TNM stage of the primary BC due to collinearity with ER status and the size of the primary tumor, respectively. In the current clinical practice, only patients with ER-positive tumors receive endocrine therapy and only patients with HER2-positive tumors receive trastuzumab; these co-occurrences were considered in the model by using composite categorical variables. A description of the studies included in the analyses is provided in **Table S2**. Follow-up started three months after invasive first primary BC diagnosis, in order to exclude synchronous CBCs, and ended at date of CBC, distant metastasis (but not at loco-regional relapse), CPM, or last date of follow-up (due to death, being lost to follow-up, or end of study), whichever occurred first. We considered that after loco-regional relapse, a woman would be still at risk for CBC as treatment for loco-regional relapse would not affect the contralateral breast cancer (CBC) unless adjuvant systemic treatment was given. Distant metastasis was considered as a competing risk because most of the patients receive systemic therapies after developing distant metastasis.

Age at first primary BC seemed to have a non-linear relationship with CBC; using splines we observed that CBC risk increased with age till around 50 years old and declined afterwards; see **Figure S1**. Therefore, we used a linear spline with a knot at 50 years in the prediction model. The use of this linear spline was a good compromise to address the non-linear relationship between CBC risk and age across the different baseline risks in all the studies, with different age distributions and selections (one study included only women aged under 50 years). Moreover, the observed non-linear relationship resembled the shape of age-related BC incidence curves with an increased risk until menopausal age followed by a decrease (Clemmensen’s hook)[6].

## *2. Multiple imputation of missing values*

The percentage of missing values across the predictors varied between 5.1% and 94.2% for morphology of first primary BC and *BRCA* mutation, respectively. In the individual patient data (IPD), both sporadic and systematic missing may occur. The former are missing values within a study, the latter are values missing for all individuals within a particular study[7-9].

For our analyses, we used ten imputed datasets based on the multiple imputation chained equations (MICE) using 50 iterations. The visit sequence of the variables was in ascending order of the number of missing values. This technique improves the accuracy and the statistical power assuming missing is at random (MAR). In the imputation procedure, we also used the year of first primary BC diagnosis since this information provides a better correlation structure among covariates used as predictors in the imputation model. Since there were systematic missing data, we used the imputation model based on the stratified multiple imputation strategy (SMI). In this approach, the variable identifying the study was used as covariate to improve substantially the imputation especially for the systematic missing predictors that might occur in the individual patient data (IPD) from multiple studies[9]. Continuous, binary and multiple categorical variables were imputed using predictive mean matching, binary and polytomous logistic regression, respectively. Time-to-event outcome defined as time to contralateral breast, time to death, and time to distant metastasis were included in the imputation process through the Nelson-Aalen cumulative hazard estimator[10]. For every variable with missing data, every imputation model selects predictors based on correlation structure underlying the data. We recoded the variables chemotherapy and morphology after imputation. In particular, information about neoadjuvant and adjuvant chemotherapy were separately imputed. Then, we created a chemotherapy variable by combining the variables for neoadjuvant and adjuvant chemotherapy in every imputed dataset. Morphology of primary tumor was imputed by keeping all original categories (‘Lobular’, ‘Ductal’, ‘Mixed’ and ‘Other’). After multiple imputation, we created two categories ‘Lobular including mixed’ and ‘Ductal including other’ to address possible overfitting due to the small samples of ‘Mixed’ and ‘Other’ categories. Since in current clinical practice, only estrogen receptor (ER) positive patients receive endocrine therapy and only human epidermal growth factor receptor 2 (HER2) positive patients receive trastuzumab, composite categorical factors of ER and endocrine therapy and of HER2 and trastuzumab therapy were considered in the model building. However, in our data, 2% of patients with 70 CBC events were coded as ER-negative treated with endocrine therapy and 0.2% of patients with 7 CBC events were coded as HER2-negative treated with trastuzumab therapy. In every imputed dataset, we recoded those patients as ER-positive treated with endocrine treatment and HER2-positive treated with trastuzumab since the largest proportion of patients (53%) were ER-positive treated with endocrine therapy and 82% were HER2-positive treated with trastuzumab in the complete data.

We used the R package mice (version 2.46.0) to impute our data and combine the estimates using Rubin’s rules.

## *3. Complete case analysis*

When a missing data pattern is completely at random (MCAR), imputation of missing data is not necessary. Therefore, descriptive analyses were performed to check whether the missing data pattern was MCAR. For completeness, the patients and first primary breast cancer characteristics and results of the multivariable subdistributional hazard model based on the case set with complete data are shown in **Table S3** and **Table S4,** respectively. The prediction performance of the risk prediction model was not investigated since in the case set with complete data all cases came from one geographic area (Western Europe) and the number of CBC event did not reach the number of events required for an external validation.

## *4. Model diagnostics and baseline recalibration*

For the multivariable model, we checked the assumption of proportional subdistribution hazards graphically using Schoenfield residuals. Heterogeneity of baseline risks between studies was taken into account using the study as a stratification term. We estimated the cumulative incidence at 5- and 10- year using the baseline hazard of the Netherlands Cancer Registry (NCR) dataset to improve the model calibration, since this is our largest cohort (67% of the data) and is based on complete incidence data thus provides a representative cumulative incidence of CBC (4.6% at 10 years). The stratified model and the application of the Rubin’s rules took into account both the between study and between imputation variation.

## *5. Leave-one-study-out cross-validation*

We used leave-one-study-out cross-validation (also known as an internal-external validation), in which a model for predicting CBC risk is developed in all studies except one whose external validity is evaluated (every study is excluded once in this process). For the studies where the number of CBC events was insufficient for external validation, we used the geographic area as a unit of splitting. For time-to-event outcomes at least 100 events per study are required for external validation[11]. The geographic area corresponding to every study is shown in **Table S5**.

We evaluated the discrimination accuracy using the time-dependent area under the curve (AUC) at 5- and 10-year. The Inverse Probability of Censoring Weighting (IPCW) was computed to estimate of cumulative/dynamic time-dependent AUCs[12]. Since the mortality and distant metastasis were competing risks, a control was defined as a subject not experiencing a CBC at 5- and 10-year, respectively. The AUC estimate and the corresponding confidence intervals were computed by bootstrapping 100 times every imputed dataset in each validation study. The AUCs and the corresponding confidence intervals were pooled using Rubin’s rules.

We did not consider delayed-entry patients (with prevalent BC) to evaluate the discrimination accuracy of the prediction models since no standard performance measures are currently available in the statistical literature to account for left-truncated follow-up time. In our study the median of delayed entry was 0.6 years.

We assessed the calibration of the models using calibration-in-the-large, calibration slope, and calibration plots per study[13]. Calibration plots report the predicted probabilities on the x-axis and the observed probabilities on the y-axis. For time-to-event data, this plot can be generated at multiple time points. To reproduce the nomogram building, we used the predicted and observed cumulative incidence of 5- and 10-year as time points for the calibration plots. The observed and predicted outcomes are divided by quartiles of predicted values. In case of good overall calibration, all points in a calibration plot are near the 45-degree line starting at the origin (0,0). If points are below the 45-degree line, models overestimate the observed risk (overfitting). If points are above the 45-degree line, the model underestimates the observed risk (underfitting). In each validation study, calibration slopes and predicted probabilities at 5 and 10 years were calculated in every imputed dataset. Then, for each validation study, calibration slopes and the predicted probabilities were pooled using Rubin’s rules. Calibration plots at 5- and 10-year are shown in **Figures S2** **and** **S3,** respectively.

## *6. Clinical utility*

The decision curve analysis combines the direct applicability of the decision-analytic methods with the mathematical simplicity of accuracy metrics[14]. The mathematical background of the net benefit calculation was originally developed by Peirce in 1884[15]. More recently, other publications expanded this work and proposed and gave emphasis why the net benefit measures should be used beyond measures of discrimination and calibration to assess the accuracy of prediction models[16].

The net benefit (NB) is calculated as:

$$NB= \frac{TP}{n}- \frac{FP}{n}\left( \frac{p_{t}}{{1-p}_{t}} \right)$$

Where n is the total sample size *TP* = true positive counts; *FP* = false positive counts; *p_t_* = risk threshold that defines the high risk and low risk patients. The ratio $\frac{p_{t}}{{1-p}_{t}}$ represents the relative weight of the harm of unnecessary contralateral preventive mastectomies (CPM) versus the benefit of CBC patients who truly need the surgery. To draw the decision curve, the net benefit is calculated for different values of *p_t_*.

The risk thresholds and the calculation of the true positives and false negatives in case of censored data with competing risks are defined as:

$$TP=\left\{ I\left( t \right) | X=1 \right\}\cdot P\left( X=1 \right)\cdot n$$

$$FP=\left\{ 1-I\left( t \right) | X=1 \right\}\cdot P\left( X=1 \right)\cdot n$$

Where:

n = total number of BC patients;

*I(t)* = cumulative incidence of CBC predicted by the prediction model at time *t*;

$$X=\left\{ \begin{aligned} 1 predicted cumulative incidence at time t \geq p_{t} \\ 0 predicted cumulative incidenceat time t < p_{t} \end{aligned} \right.$$

More mathematical details have been provided by Vickers in 2008 and Kerr in 2016[17, 18].

The landmark time *t* was set to 5 and 10 years since the prediction model provided the estimated cumulative incidence at 5 and 10 years.

Although discrimination measures such as sensitivity, specificity, Area Under the Curve (AUC), and c-statistic and calibration measures cannot be used to assess the clinical utility of a prediction model, net benefit is larger for more discriminating models and decrease with poor calibration[19]. Referring to our model, the reduction in the number of unnecessary CPM per 1,000 patients without a decrease in the number of patients who correctly received the surgery is calculated as:

$(net benefit of the model - net benefit of treat all)/( pt /(1- pt)) \times1,00$0

For example, at a risk threshold of 10% the difference between the net benefit of the prediction model and the net benefit of treat all was 0.0179, the number of avoidable unnecessary CPM would be [0.0179/(0.10/0.90)] = 0.1611 x 1000 = 161.1 per 1,000 patients.

Results of the decision curve analysis that were not reported in Table 2, are reported in **Table S7.** The utilization of 5-year CBC risk prediction in terms of net benefit showed that for some risk thresholds (between 1.5–4.5%), the prediction model might be clinically useful to avoid unnecessary CPM among *BRCA1* patients and to counsel necessary CPM among non-carriers. As an example, if a clinician finds it acceptable to perform around 21 unnecessary CPM to prevent one CBC (one necessary CPM), a risk threshold of 4.5% may be used to define high and low risk *BRCA1/2* patients based on the absolute 5-year CBC risk prediction estimated by the model. In this scenario, approximately 163 CPMs per 1,000 patients may be avoided using the model compared to counseling CPM to all *BRCA1/2* carriers. Similarly, if unnecessarily performing a CPM in 39 patients would be acceptable to prevent one CBC, a risk threshold of 2.5% may be used to define high and low risk non-carriers; and this would include around necessary 491 CPMs per 1,000 patients. The decision curves in **Figures S4** provide a comprehensive overview of the net benefit for a range of harm-benefit thresholds at 5-year CBC risk.

## *7. Formula to estimate the contralateral breast cancer risk*

Our developed model is a subdistributional proportional hazard Fine and Gray model. The estimated cumulative incidence of CBC was estimated using the following formula:

$$F\left( t \right)=1-\left\{ \left[ S_{0}(t) \right]^{exp(\boldsymbol{LP})} \right\}$$

Where *t* is the time (in years) since primary BC, $F\left( t \right)$ is the cumulative incidence of CBC and $S_{0}(t)$ is the probability to survive beyond for baseline covariate values. The baseline survival estimates according to the model and time are:

$$S_{0}\left( 5 \right)= 0.984$$

$$S_{0}\left( 10 \right)= 0.968$$

And

*Linear Predictor* *(LP)* =

– 0.223 + 0.007× Age – 0.023 × Age’ + 0.303 × I[Family history = Yes] + 1.304 × I[*BRCA = BRCA1*]

+ 0.941 × I[*BRCA = BRCA2*] – 0.142 × I[Nodal status = positive] – 0.047 × I[Size of PBC = (2,5] *cm*]

+ 0.128 × I[Size of PBC = greater than 5 *cm*] + 0.209 × I[Morphology of PBC = lobular including mixed]

– 0.120× I[Grade of PBC = moderately differentiated] – 0.291 × I[Grade of PBC = poorly/undifferentiated]

– 0.266 × I[Chemotherapy = yes] + 0.009 × I[Radiotherapy to the breast = yes] + 0.356 × I[ER-negative without endocrine therapy ] + 0.559 × I[ER-positive without endocrine therapy ]+ 0.082 × I[HER2-negative without trastuzumab] – 0.005 × I[HER2-positive without trastuzumab]

Where Age’ = max(Age – 50, 0)

## *8. Results of the prediction model without BRCA mutation*

Because a patient may not have been tested for the *BRCA* gene mutations, this information may not be available before or on the day of first primary BC diagnosis or treatment decisions. Moreover, information about *BRCA* mutations was largely missing in the databases we used. Thus, we also developed and validated a CBC prediction model without *BRCA* mutations to also provide an individualized risk prediction tool for patients not tested. Results of the risk prediction model in terms of relative subdistributional hazard ratio (sHRSs) and the corresponding 95% confidence intervals (CI) for patients not tested for *BRCA* gene mutations are provided in **Table S8**.

The assessments of prediction performance are shown in **Figures S5, S6, and S7.** The discrimination accuracy at 5 years was 0.59 (95% CI: 0.54 – 0.63; 95% prediction interval (PI): 0.46 – 0.71) and at 10 years was 0.59 (95% CI: 0.56 – 0.62; 95% PI: 0.52 – 0.66), as shown in **Figure S5**. The calibration-in-the-large was -0.17 (95% CI: -0.72 – 0.38; 95% PI: -1.70– 1.36), as shown in the **Figure S5 panel C;** and calibration slope was 0.81 (95% CI: 0.63 – 0.99; 95% PI: 0.50 – 1.12) in the leave-one-study-out cross-validation, as shown in **Figure S5 panel D**. The calibration plots at 5- and 10-year are reported in **Figures S6 and S7**, respectively.

The utilization of 10-year CBC risk prediction in terms of net benefit showed that for some risk thresholds (between 3.5–5.5%), the prediction model might be clinically useful to avoid unnecessary CPM among patients with family history and to counsel necessary CPM among patients without first-degree relatives with BC. For example, if a clinician finds it acceptable to perform around 21 unnecessary CPM to prevent one CBC, a risk threshold of 4.5% may be used to define high and low risk patients with family history based on the absolute 10-year CBC risk prediction estimated by the model. In this scenario, 55 CPM per 1,000 patients may be avoided using the model compared to counseling CPM to all patients with family history; see **Table S10** and **Figure S8**. The density distribution of the estimated 10-year CBC risk prediction was shown for patients with and without family history. The overlap between the two distributions reflects that a prediction model is useful to define high and low risk patients to counsel necessary CPM and avoid unnecessary surgeries in a setting where *BRCA1/2* mutations are not tested. The decision curves in **Figures S9 and S10** provide a comprehensive overview of the net benefit for a range of harm-benefit thresholds.

## *9. Formula to estimate the contralateral breast cancer risk in patients not tested for BRCA*

The formula for the alternative model is reported below. Baseline survival estimates according to the model and time are:

$$S_{0}\left( 5 \right)= 0.982$$

$$S_{0}\left( 10 \right)= 0.965$$

And

*Linear Predictor (LP)* =

+ 0.108 – 0.002 × Age – 0.018 × Age’ + 0.473 × I[Family history = Yes] – 0.143 × I[Nodal status = positive] – 0.041 × I[Size of PBC = (2,5] *cm*] + 0.108 × I[Size of PBC = greater than 5 *cm*] + 0.181 × I[Morphology of PBC = lobular including mixed] – 0.032 × I[Grade of PBC = moderately differentiated] – 0.135 × I[Grade of PBC = poorly/undifferentiated]– 0.248 × I[Chemotherapy = yes] – 0.034 × I[Radiotherapy to the breast = yes] + 0.522 × I[ER-negative without endocrine therapy] + 0.592 × I[ER-positive without endocrine] + 0.232 × I[HER2-negative without trastuzumab] + 0.082 × I[HER2-positive without trastuzumab]

Where Age’ = max(Age – 50, 0)

## *10. Assessment of limited information of contralateral preventive mastectomy (CPM)*

Information about CPM was not available in most studies. This lack of information may underestimate the cumulative CBC incidence because patients underwent CPM should not be considered to be at risk to develop CBC, though a small proportion of 1.3% of CBC was observed after CPM among *BRCA1* or *BRCA2-*related breast cancer patients[20]. We investigated the impact of CPM on CBC cumulative incidence estimation in the BOSOM and EMC datasets, in which this information was complete and CPM was not within 3 months after first BC diagnosis, i.e. for 3,760 out of 3,793 and 3,390 out of 3,398, respectively. In these two studies, we compared the estimated cumulative incidence curves in which we applied censoring for CPM or considering in the risk set patients experiencing CPM at first primary BC or during the follow-up.

**Figure S11** shows the cumulative incidence estimation of the two scenarios. As expected, the cumulative incidence was underestimated when we ignored the occurrence of CPM. However, there was only a small difference between the two curves: the estimated cumulative incidence at 10 years was 5.6% (95% CI: 4.9 – 6.4%) considering CPM, and 5.3% (4.6 – 6.0%) not considering CPM in the BOSOM dataset; and 5.7% (5.0 – 6.6%) considering CPM, and 5.6% (4.8 – 6.4%) not considering CPM in the EMC dataset. Therefore, although the CPM was not available for most studies, we concluded that the cumulative incidence of CBC was only slightly underestimated due to missing CPM information.

## *References*

1. Michailidou K, Lindstrom S, Dennis J, Beesley J, Hui S, Kar S, Lemacon A, Soucy P, Glubb D, Rostamianfar A *et al*: **Association analysis identifies 65 new breast cancer risk loci**. *Nature* 2017, **551**(7678):92-94.

2. Schmidt MK, Tollenaar RA, de Kemp SR, Broeks A, Cornelisse CJ, Smit VT, Peterse JL, van Leeuwen FE, Van't Veer LJ: **Breast cancer survival and tumor characteristics in premenopausal women carrying the CHEK2*1100delC germline mutation**. *J Clin Oncol* 2007, **25**(1):64-69.

3. Schmidt MK, van den Broek AJ, Tollenaar RA, Smit VT, Westenend PJ, Brinkhuis M, Oosterhuis WJ, Wesseling J, Janssen-Heijnen ML, Jobsen JJ *et al*: **Breast Cancer Survival of BRCA1/BRCA2 Mutation Carriers in a Hospital-Based Cohort of Young Women**. *J Natl Cancer Inst* 2017, **109**(8).

4. Font-Gonzalez A, Liu L, Voogd AC, Schmidt MK, Roukema JA, Coebergh JW, de Vries E, Soerjomataram I: **Inferior survival for young patients with contralateral compared to unilateral breast cancer: a nationwide population-based study in the Netherlands**. *Breast Cancer Res Treat* 2013, **139**(3):811-819.

5. Kramer I, Schaapveld M, Oldenburg HSA, Sonke GS, McCool D, Van Leeuwen FE, van de Vijver KK, Russell NS, Linn SC, Siesling S *et al*: **The influence of adjuvant systemic regimens on contralateral breast cancer risk and receptor subtype**. *J Natl Cancer Inst* In press.

6. Bouchardy C, Usel M, Verkooijen HM, Fioretta G, Benhamou S, Neyroud-Caspar I, Schaffar R, Vlastos G, Wespi Y, Schafer P *et al*: **Changing pattern of age-specific breast cancer incidence in the Swiss canton of Geneva**. *Breast Cancer Res Treat* 2010, **120**(2):519-523.

7. Riley RD, Lambert PC, Abo-Zaid G: **Meta-analysis of individual participant data: rationale, conduct, and reporting**. *BMJ* 2010, **340**:c221.

8. Resche-Rigon M, White IR, Bartlett JW, Peters SA, Thompson SG, Group P-IS: **Multiple imputation for handling systematically missing confounders in meta-analysis of individual participant data**. *Stat Med* 2013, **32**(28):4890-4905.

9. Jolani S, Debray TP, Koffijberg H, van Buuren S, Moons KG: **Imputation of systematically missing predictors in an individual participant data meta-analysis: a generalized approach using MICE**. *Stat Med* 2015, **34**(11):1841-1863.

10. White IR, Royston P: **Imputing missing covariate values for the Cox model**. *Stat Med* 2009, **28**(15):1982-1998.

11. Collins GS, Ogundimu EO, Altman DG: **Sample size considerations for the external validation of a multivariable prognostic model: a resampling study**. *Stat Med* 2016, **35**(2):214-226.

12. Blanche P, Dartigues JF, Jacqmin-Gadda H: **Estimating and comparing time-dependent areas under receiver operating characteristic curves for censored event times with competing risks**. *Stat Med* 2013, **32**(30):5381-5397.

13. Snell KI, Hua H, Debray TP, Ensor J, Look MP, Moons KG, Riley RD: **Multivariate meta-analysis of individual participant data helped externally validate the performance and implementation of a prediction model**. *J Clin Epidemiol* 2016, **69**:40-50.

14. Vickers AJ, Elkin EB: **Decision curve analysis: a novel method for evaluating prediction models**. *Med Decis Making* 2006, **26**(6):565-574.

15. Peirce CS: **The numerical measure of the success of predictions**. *Science* 1884, **4**(93):453-454.

16. Localio AR, Goodman S: **Beyond the usual prediction accuracy metrics: reporting results for clinical decision making**. *Ann Intern Med* 2012, **157**(4):294-295.

17. Vickers AJ, Cronin AM, Elkin EB, Gonen M: **Extensions to decision curve analysis, a novel method for evaluating diagnostic tests, prediction models and molecular markers**. *BMC Med Inform Decis Mak* 2008, **8**:53.

18. Kerr KF, Brown MD, Zhu K, Janes H: **Assessing the Clinical Impact of Risk Prediction Models With Decision Curves: Guidance for Correct Interpretation and Appropriate Use**. *J Clin Oncol* 2016, **34**(21):2534-2540.

19. Van Calster B, Vickers AJ: **Calibration of risk prediction models: impact on decision-analytic performance**. *Med Decis Making* 2015, **35**(2):162-169.

20. van Sprundel TC, Schmidt MK, Rookus MA, Brohet R, van Asperen CJ, Rutgers EJ, Van't Veer LJ, Tollenaar RA: **Risk reduction of contralateral breast cancer and survival after contralateral prophylactic mastectomy in BRCA1 or BRCA2 mutation carriers**. *Br J Cancer* 2005, **93**(3):287-292.

# Supplementary Tables

| **Table S1.** Data source flowchart. | | | | | |
| --- | --- | --- | --- | --- | --- |
|  | Source of data | | | | |
|  | ABCS | BCAC^‡^ | BOSOM | EMC | NCR |
| Number of patients | 2,390 | 182,898 | 7,105 | 3,483 | 94,600 |
|  |  |  |  |  |  |
| Eligibility criteria, *number of patients excluded* |  |  |  |  |  |
| Studies from Asian countries | - | 7,348 | - | - | - |
| Patients of non-European descent | - | 46,670 | - | - | - |
| Year of PBC diagnosis before 1990 | - | 3,358 | 3,126 | - | - |
| Year of PBC diagnosis missing | - | 26,291 | - | - | - |
| PBC stage 0 | 122 | 34 | 2 | - | - |
| PBC stage IV | 94 | 1,675 | 104 | - | 4,569 |
| Patients did not undergo surgery | 24 | 1,138 | 43 | 5 | 5,174 |
| Number of eligible patients | 2,150 | 96,384 | 3,830 | 3,478 | 84,857 |
|  |  |  |  |  |  |
| No follow-up or follow-up less than 3 months | 171 | 13,144 | 70 | 88 | 1,719 |
| Familiar breast cancer studies | - | 4,635 | - | - | - |
| Studies with less than 10 CBC events | - | 38,116 | - | - | - |
| Number of patients included in the analysis (number of patients with CBC) | 1,979 (19) | 40,489 (707) | 3,760 (288) | 3,390  (221) | 83,138 (3,447) |
|  |  |  |  |  |  |
| Total number of patients included in the analysis  (number of CBC) | 132,756  (4,682 of which 3,974 invasive and 708 *in-situ*) | | | | |
|  |  |  |  |  |  |
| Abbreviations: ABCS: Amsterdam Breast Cancer Study BCAC: Breast Cancer Association Consortium. ^‡^BCAC is composed of 102 studies world-wide. The 40,489 patients selected for the analysis came from 16 studies.  BOSOM: Breast Cancer Outcome Study of Mutation carriers EMC: Erasmus Medical Center  NCR: Netherlands Cancer Registry PBC: primary breast cancer CBC: contralateral breast cancer | | | | | |
|  |  |  |  |  |  |
|  |  |  |  |  |  |
|  |  |  |  |  |  |

**Table S2**: see additional file 2: Table S2

**Table S3:** Patients and first primary breast cancer characteristics used in the contralateral breast cancer risk prediction model in the complete case and all case analyses.

|  |  | |
| --- | --- | --- |
| **Factors at primary breast cancer** | **N** | **%** |
|  | **132,756** | **100.0** |
| Age, *years* |  |  |
| Median (range) | 57 (18 - 101) | |
| Missing | - | |
| Family history |  |  |
| Yes | 5,959 | 19.5 |
| No | 24,582 | 80.5 |
| Missing | 102,215 | - |
| *BRCA* mutation |  |  |
| *BRCA1* | 333 | 4.3 |
| *BRCA2* | 167 | 2.2 |
| Non carrier | 7,204 | 93.5 |
| Missing | 125,052 | - |
| Nodal status |  |  |
| Positive | 48,979 | 39.1 |
| Negative | 76,356 | 60.9 |
| Missing | 7,421 | - |
| Tumor size, *cm* |  |  |
| ≤ 2 | 75,849 | 60.8 |
| (2-5] | 43,075 | 34.5 |
| > 5 | 5,916 | 4.7 |
| Missing | 7,916 | - |
| Tumor grade |  |  |
| well differentiated | 25,271 | 21.7 |
| moderately differentiated | 53,385 | 45.7 |
| poorly/undifferentiated | 38,045 | 32.6 |
| Missing | 16,055 | - |
| ER status |  |  |
| Positive | 97,460 | 80.5 |
| Negative | 23,625 | 19.5 |
| Missing | 11,671 | - |
| HER2 status |  |  |
| Positive | 15,401 | 17.4 |
| Negative | 72,891 | 82.6 |
| Missing | 44,464 | - |
| Morphology |  |  |
| Ductal | 96,561 | 76.6 |
| Lobular | 14,681 | 11.7 |
| Mixed | 4,982 | 4.0 |
| Other | 9,780 | 7.8 |
| Missing | 6,752 | - |
| Adjuvant chemotherapy |  |  |
| Yes | 46,868 | 38.2 |
| No | 75,785 | 61.8 |
| Missing | 10,103 | - |
| Neoadjuvant chemotherapy |  |  |
| Yes | 7,213 | 6.0 |
| No | 112,267 | 94.0 |
| Missing | 13,276 | - |
| Endocrine adjuvant therapy |  |  |
| Yes | 65,959 | 54.1 |
| No | 56,055 | 45.9 |
| Missing | 10,742 | - |
| Trastuzumab adjuvant therapy |  |  |
| Yes | 6,875 | 6.7 |
| No | 9,6324 | 93.3 |
| Missing | 29,557 | - |
| Radiation in the breast |  |  |
| Yes | 85,029 | 69.5 |
| No | 37,237 | 30.5 |
| Missing | 10,490 | - |
| CBC cumulative incidence, *%* |  |  |
| 5-year (95%CI) | 2.1 (2.1 - 2.2) | |
| 10-year (95%CI) | 4.1 (4.0 - 4.3) | |
| Abbreviations:  PBC: primary breast cancer; ER: estrogen-receptor; HER2: human epidermal growth factor receptor 2; CBC: contralateral breast cancer; CI: confidence interval; | | |

**Table S4:** Results of multivariable subdistributional hazard model using the complete case dataset.

|  | | |
| --- | --- | --- |
| **Factor (categories) at primary breast cancer** | **Multivariable analysis** | |
|  | **sHR** | **95% CI** |
| Age, *years* | 1.48^a^ | 0.73 – 3.00^a^ |
| Family history (yes versus no) | 1.36 | 0.69 - 2.70 |
| *BRCA* mutation |  |  |
| *BRCA1* versus non-carrier | 5.28 | 2.13 - 13.10 |
| *BRCA2* versus non-carrier | 2.30 | 0.50 - 10.51 |
| Nodal status (positive versus negative) | 1.37 | 0.56 - 3.34 |
| Tumor size, *cm* |  |  |
| (2,5] versus ≤ 2 | 0.57 | 0.22 - 1.47 |
| > 5 versus ≤ 2 | 3.53 | 1.10 - 11.34 |
| Morphology (lobular including mixed versus ductal including other) | 0.99 | 0.33 - 2.88 |
| Grade |  |  |
| Moderately differentiated versus well differentiated | 0.91 | 0.28 - 2.88 |
| Poorly differentiated versus well differentiated | 0.84 | 0.23 - 3.04 |
| Chemotherapy (yes versus no) | 0.38 | 0.16 – 0.89 |
| Radiotherapy to the breast (yes versus no) | 1.26 | 0.56 - 2.83 |
| ER (positive or negative) / endocrine therapy (yes or no) |  |  |
| negative/no versus positive/yes | 1.42 | 0.53 - 3.77 |
| positive/no versus positive/yes | 2.38 | 0.90 - 6.31 |
| HER2 (positive or negative) / trastuzumab therapy (yes or no) |  |  |
| negative/no versus positive/yes | 0.71 | 0.22 - 2.36 |
| positive/no versus positive/yes | 0.32 | 0.07 - 1.46 |
| Abbreviations: sHR: subdistributional hazard ratio; CI: confidence interval; ER: estrogen receptor; HER2: human epidermal growth factor receptor 2; ^a^ Age was parameterized as a linear spline at 50. For presentation purposes, we here provide the sHR for the 75^th^ versus the 25^th^ percentile. | | |

**Table S5:** List of BCAC studies (including ABCS source) with the corresponding country and geographic area. For studies in which the number of contralateral breast cancer events was insufficient for external validation, the geographic area was used.

| **Study** | **Country** | **Geographic area or study** |
| --- | --- | --- |
| ABCS | Netherlands | Europe - Other |
| ABCFS | Australia | United States and Australia |
| BBCC | Germany | Europe - Other |
| CGPS | Denmark | Europe - Scandinavia |
| HEBCS | Finland | Europe - Scandinavia |
| KARBAC | Sweden | Europe - Scandinavia |
| KARMA | Sweden | Europe - Scandinavia |
| LMBC | Belgium | Europe - Other |
| MARIE | Germany | Europe - Other |
| MEC | United States | United States and Australia |
| ORIGO | Netherlands | Europe - Other |
| PBCS | Poland | Europe - Other |
| PKARMA | Sweden | Europe - Scandinavia |
| POSH | United Kingdom | Europe - United Kingdom |
| SEARCH | United Kingdom | Europe - United Kingdom |
| SKKDKFZS | Germany | Europe - Other |
| SZBCS | Poland | Europe - Other |
|  | | |

**Table S6**: see Additional file 3: Table S6 (Patient and primary breast cancer characteristics per study).

**Table S7**: Clinical utility of the 5-year contralateral breast cancer risk prediction model. At the same probability threshold, the net benefit is exemplified in *BRCA1/2* mutation carriers (for avoiding unnecessary CPM) and non-carriers (performing necessary CPM).

|  | | | | | |
| --- | --- | --- | --- | --- | --- |
| Probability threshold p_t_ (%) | Unnecessary CPMs needed to prevent a CBC^*^ | *BRCA1/2* mutation carriers | | Non-carriers | |
|  |  | Net benefit  versus  treat all patients with CPM  (per 1000) | Avoided unnecessary CPMs per 1000 patients | Net benefit  versus  treat none  (per 1000) | Performed necessary CPMs per 1000 patients |
| 1.5 | 65.7 | 0.0 | 0.0 | 3.3 | 216.7 |
| 2.5 | 39.0 | 0.1 | 3.9 | 12.6 | 491.4 |
| 3.5 | 27.6 | 2.3 | 63.4 | 0.1 | 2.8 |
| 4.5 | 21.2 | 7.7 | 163.4 | 0.0 | 0.0 |
| CPM: contralateral preventive mastectomy; CBC: contralateral breast cancer; ^*^ The number of unnecessary contralateral mastectomies needed to prevent a CBCis calculated by:  (1-p_t_)/p_t_ | | | | | |

**Table S8:** Results of multivariable subdistributional hazard model for breast cancer patients without *BRCA* mutations.

|  | | |
| --- | --- | --- |
| **Factor (category) at primary breast cancer** | **Multivariable analysis** | |
|  | **sHR** | **95% CI** |
| Age, *years* | 0.61^a^ | 0.56-0.66^a^ |
| Family history (yes versus no) | 1.60 | 1.50 - 1.71 |
| Nodal status(positive versus negative) | 0.87 | 0.80 - 0.93 |
| Tumor size, *cm* |  |  |
| (2,5] versus ≤ 2 | 0.96 | 0.89 - 1.03 |
| > 5 versus ≤ 2 | 1.11 | 0.97 - 1.28 |
| Morphology (lobular including mixed versus ductal including other) | 1.20 | 1.10 - 1.30 |
| Grade |  |  |
| Moderately differentiated versus well differentiated | 0.97 | 0.90 - 1.04 |
| Poorly differentiated versus well differentiated | 0.87 | 0.79 - 0.96 |
| Chemotherapy (yes versus no) | 0.78 | 0.71 - 0.85 |
| Radiation of the breast (yes versus no) | 0.97 | 0.90 - 1.03 |
| ER (positive or negative) / endocrine therapy (yes or no) |  |  |
| negative/no versus positive/yes | 1.67 | 1.54 - 1.84 |
| positive/no versus positive/yes | 1.81 | 1.67 - 1.96 |
| HER2 (positive or negative) / trastuzumab therapy (yes or no) |  |  |
| negative/no versus positive/yes | 1.26 | 1.08 - 1.48 |
| positive/no versus positive/yes | 1.08 | 0.91 - 1.30 |
| Abbreviations: sHR: subdistributional hazard ratio; CI: confidence interval; ER: estrogen receptor; HER2: human epidermal growth factor receptor 2; ^a^: Age was parameterized as a linear spline at 50. For representation purposes, we here provide the sHR for the 75th versus the 25th percentile. | | |

**Table S9.** Clinical utility of the 5-year contralateral breast cancer risk prediction model in non-*BRCA* tested patients. At the same probability threshold, the net benefit is exemplified in patients with family history (for avoiding unnecessary CPM) and patients without family history (performing necessary CPM).

|  | | | | | |
| --- | --- | --- | --- | --- | --- |
| Probability threshold p_t_ (%) | Unnecessary CPMs needed to prevent a CBC^*^ | Family history | | No family history | |
|  |  | Net benefit  versus  treat all patients with CPM  (per 1000) | Avoided unnecessary CPMs per 1000 patients | Net benefit  versus  treat none  (per 1000) | Performed necessary CPMs per 1000 patients |
| 2.0 | 49.0 | 0.4 | 19.6 | 2.1 | 102.9 |
| 2.5 | 39.0 | 2.9 | 113.1 | 1.2 | 46.8 |
| 3.0 | 32.3 | 0.0 | 0.0 | 0.2 | 6.5 |
| CPM: contralateral preventive mastectomy; CBC: contralateral breast cancer; ^*^ The number of unnecessary contralateral preventive mastectomies needed to prevent a CBC is calculated by: (1-p_t_)/p_t_ | | | | | |

**Table S10.** Clinical utility of the 10-year contralateral breast cancer risk prediction model in non-*BRCA* tested patients. At the same probability threshold, the net benefit is exemplified in patients with family history (for avoiding unnecessary CPM) and patients without family history (performing necessary CPM).

|  | | | | | |
| --- | --- | --- | --- | --- | --- |
| Probability threshold p_t_ (%) | Unnecessary CPMs needed to prevent a CBC^*^ | Family history | | No family history | |
|  |  | Net benefit  versus  treat all patients with CPM  (per 1000) | Avoided unnecessary CPMs per 1000 patients | Net benefit  versus  treat none  (per 1000) | Performed necessary CPMs per 1000 patients |
| 3.5 | 27.6 | 0.3 | 8.3 | 5.0 | 137.9 |
| 4.5 | 21.2 | 2.6 | 55.2 | 3.0 | 63.7 |
| 5.5 | 17.2 | 8.2 | 140.9 | 1.1 | 18.9 |
| CPM: contralateral mastectomy; CBC: contralateral breast cancer; ^*^ The number of unnecessary contralateral preventive mastectomies needed to prevent a CBC is calculated by: (1-p_t_)/p_t_ | | | | | |

# Supplementary Figures


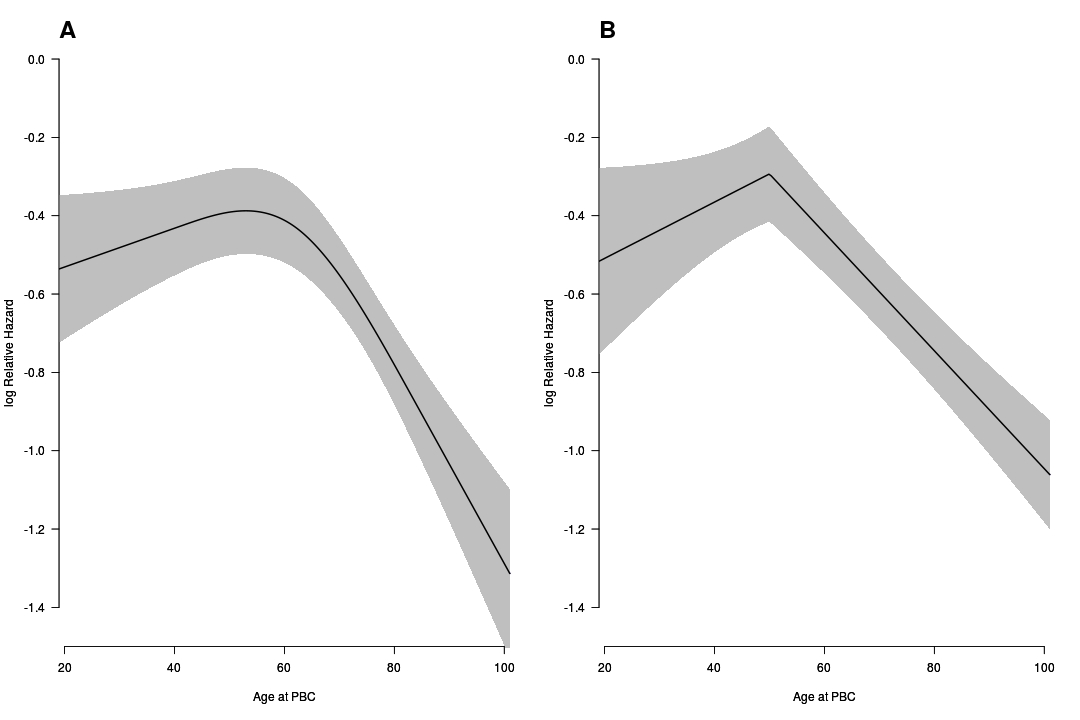


**Figure S1**: Graphical assessment of non-linear relationship of age with contralateral breast cancer risk.

A non-linear relationship between age at first primary breast cancer (x-axis) and the log relative hazard of contralateral breast cancer (y-axis) is shown. Panel A shows a restricted cubic spline with three knots. Panel B shows a linear spline with one knot located at 50 years. The curve gray area indicates the corresponding 95% confidence intervals. Both curves were estimated from a multivariable subdistributional hazard model adjusted for the variables used for the risk prediction considering death for any causes and distant metastasis as a competing risk.


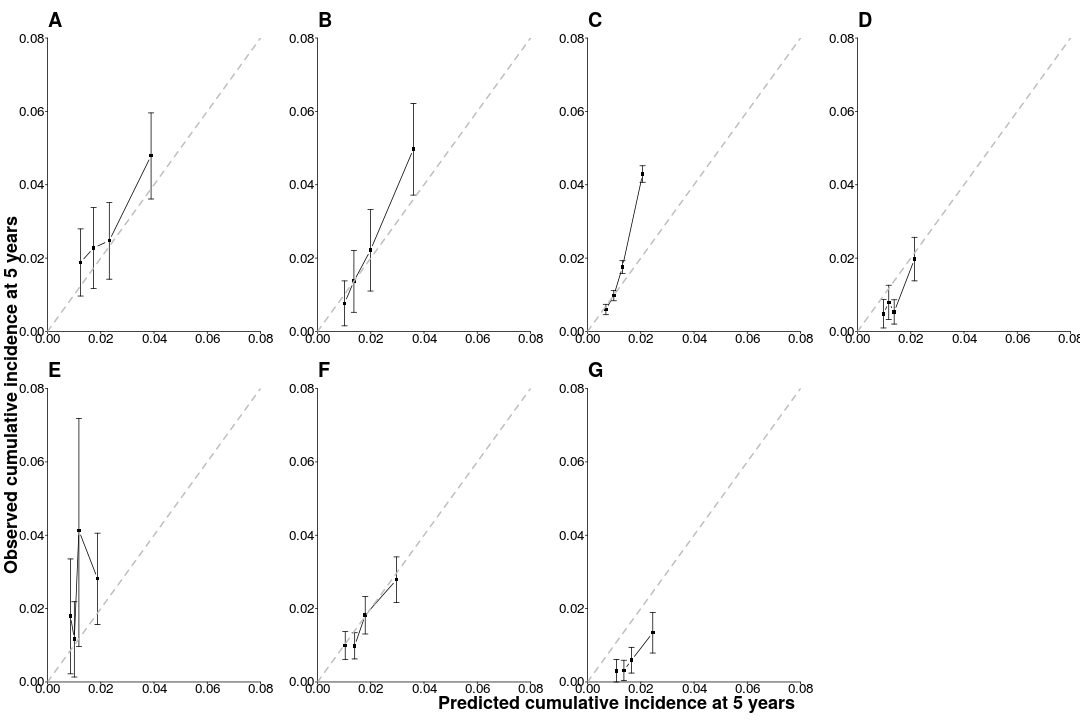


**Figure S2:** Visual assessment of calibration through calibration plots in the internal-external cross-validation at 5 years for the contralateral breast cancer risk model with *BRCA* mutation information.

The x-axis represents the predicted cumulative incidence of contralateral breast cancer at 5 years and the y-axis the observed cumulative incidence at 5 years. The black dots indicate the calibration for quartiles of predicted values. Vertical black bars indicate the 95% confidence intervals. The dashed gray line indicates perfect overall calibration. Each panel indicates a validation in one of the datasets. Panel A: Netherlands - BOSOM; Panel B: Netherlands - EMC; Panel C: Netherlands - NCR; Panel D: Europe – Scandinavia; Panel E: United States and Australia; Panel F: Europe – Other; Panel G: Europe – United Kingdom.


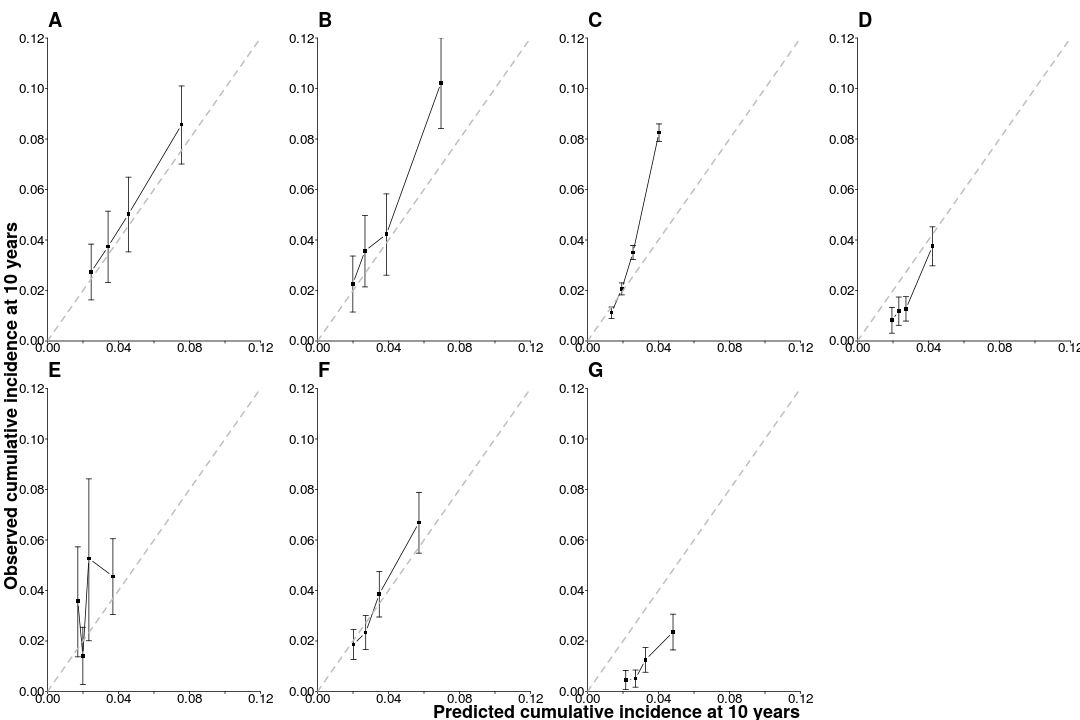


**Figure S3:** Visual assessment of calibration through calibration plots in the internal-external cross-validation at 10 years for the contralateral breast cancer risk model with *BRCA* mutation information.

The x-axis represents the predicted cumulative incidence of contralateral breast cancer at 10 years and the y-axis the observed cumulative incidence at 10 years. The black dots indicate the calibration for quartiles of predicted values. Vertical black bars indicate the 95% confidence intervals. The dashed gray line indicates perfect overall calibration. Each panel indicates a validation in one of the datasets. Panel A: Netherlands - BOSOM; Panel B: Netherlands - EMC; Panel C: Netherlands - NCR; Panel D: Europe – Scandinavia; Panel E: United States and Australia; Panel F: Europe – Other; Panel G: Europe – United Kingdom.


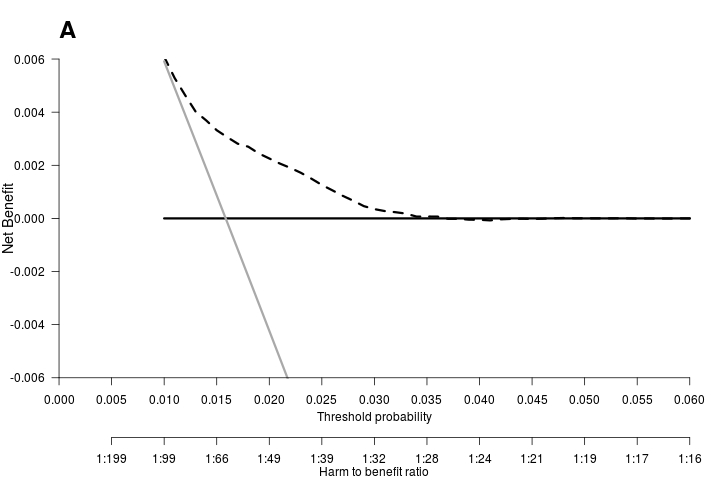

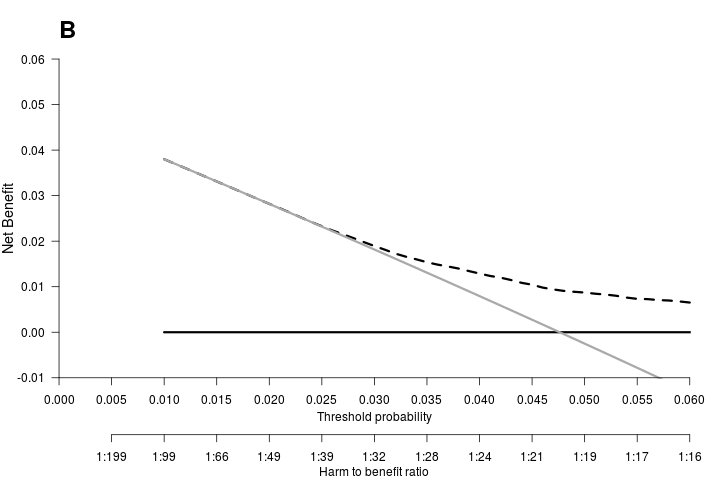


**Figure S4:** Decision curve analysis at 5 years for the contralateral breast cancer risk model including *BRCA1/2* mutation information.

Panel A shows the decision curve to determine the net benefit of the estimated 5-year predicted contralateral breast cancer (CBC) cumulative incidence for patients without a *BRCA1/2* gene mutation using the prediction model (dotted black line) compared to not treating any patients with contralateral preventive mastectomy (CPM) (black solid line). Panel B shows the decision curve to determine the net benefit of the estimated 5-year predicted CBC cumulative incidence for *BRCA1/2* mutation carriers using the prediction model (dotted black line) versus treating (or at least counseling) all patients (grey solid line). The y-axis measures net benefit, which is calculated by summing the benefits (true positives, i.e., patients with a CBC who needed a CPM) and subtracting the harms (false positives, i.e., patients with CPM who do not need it). The latter are weighted by a factor related to the relative harm of a non-prevented CBC versus an unnecessary CPM. The factor is derived from the threshold probability to develop a CBC at 5 years at which a patient would opt for CPM (e.g. 4.5%). The x-axis represents the threshold probability. Using a threshold probability of 4.5% implicitly means that CPM in 22 patients of whom one would develop a CBC if untreated is acceptable (21 unnecessary CPMs, harm to benefit ratio 1:21).


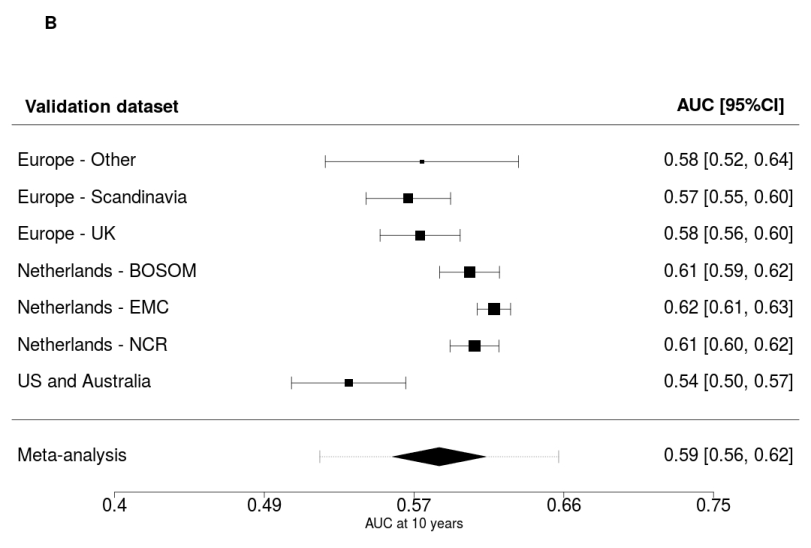

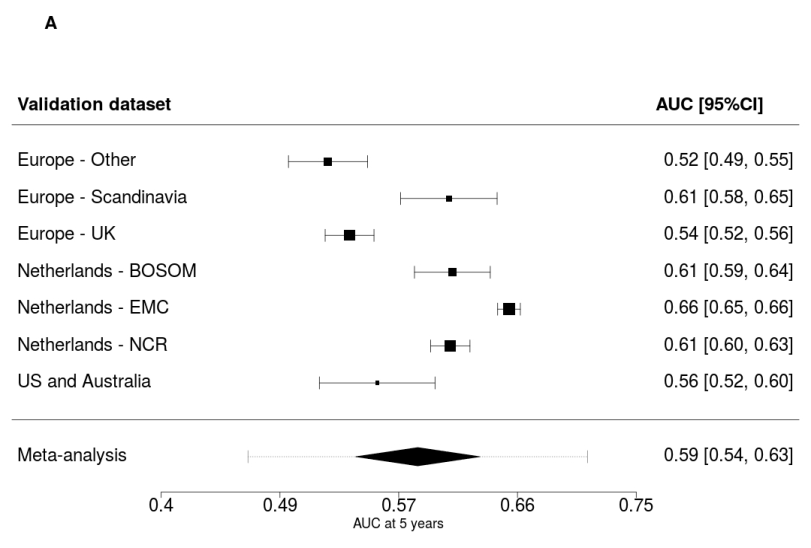

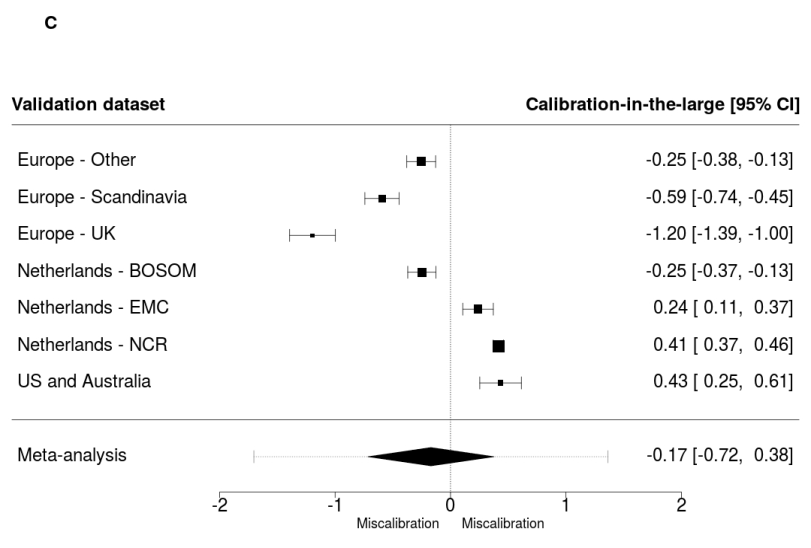

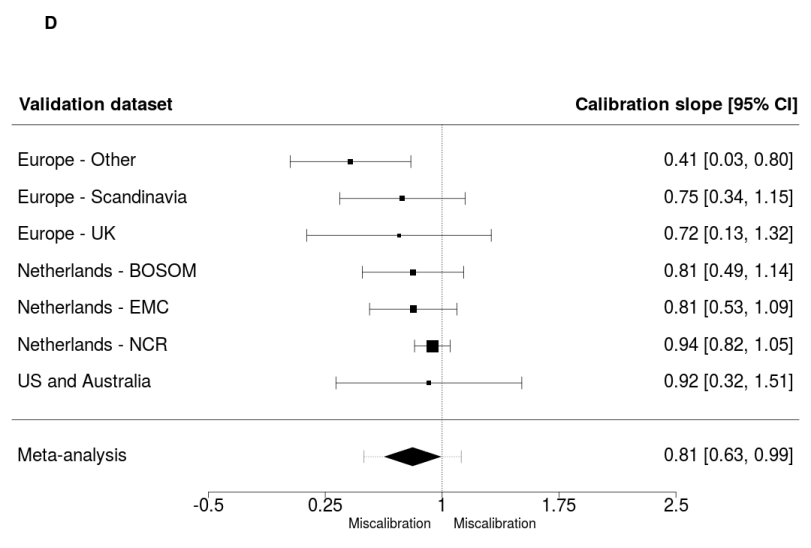


**Figure S5**: Results of the leave-one-study-out cross-validation for the contralateral breast cancer risk model at 5 and 10 years without *BRCA* mutation information.

Panel A and B show the discrimination accuracy assessed by a time-dependent AUC at 5 and 10 years, respectively. Panel C shows the calibration accuracy measured with calibration in-the-large. Panel D shows the calibration accuracy measured with calibration slope. The black squares indicate the estimated accuracy of the model in a single new validation study or geographic area. The black horizontal lines interval indicate the corresponding 95% confidence intervals of the estimated accuracy (interval whiskers). The black diamonds indicate the mean with the corresponding 95% confidence interval of the predictive accuracy and the dashed horizontal lines indicate the corresponding 95% prediction intervals.

**
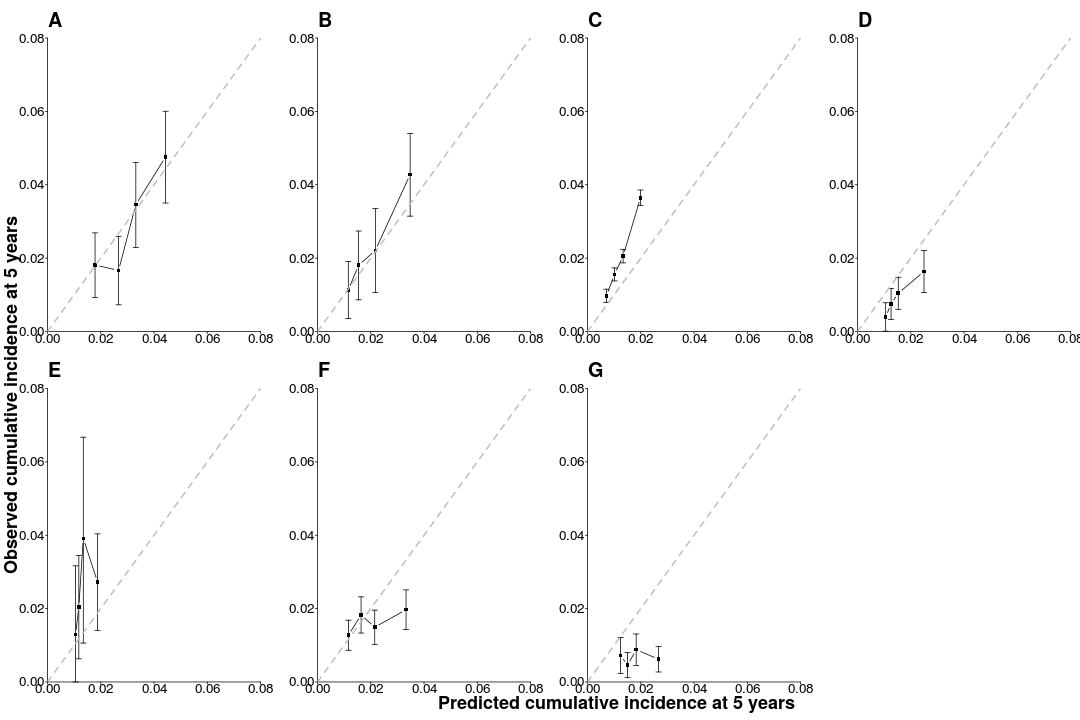
**

**Figure S6:** Visual assessment of calibration through calibration plots in the internal-external cross-validation at 5 years for the contralateral breast cancer risk model without *BRCA* gene mutation information.

The x-axis represents the predicted cumulative incidence of contralateral breast cancer at 5 years and the y-axis the observed cumulative incidence at 5 years. The black dots indicate the calibration for quartiles of predicted values. Vertical black bars indicate the 95% confidence intervals. The dashed gray line indicates perfect overall calibration. Each panel indicates a validation in one of the datasets. Panel A: Netherlands - BOSOM; Panel B: Netherlands - EMC; Panel C: Netherlands - NCR; Panel D: Europe – Scandinavia; Panel E: United States and Australia; Panel F: Europe – Other; Panel G: Europe – United Kingdom.


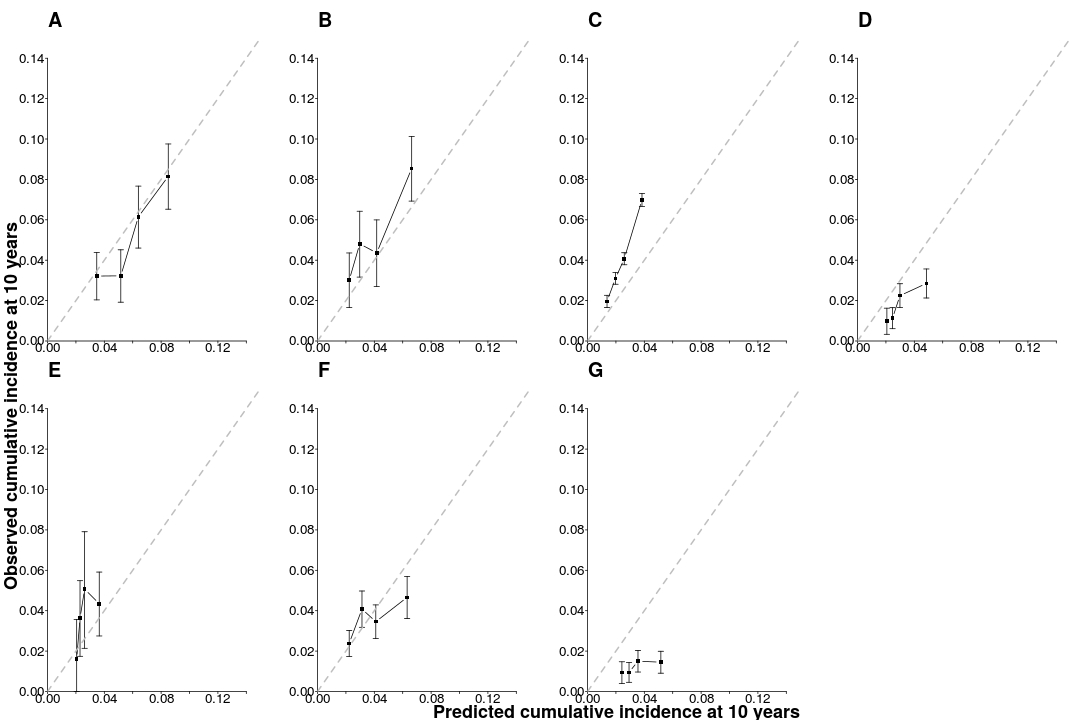


**Figure S7:** Visual assessment of calibration through calibration plots in the internal-external cross-validation at 10 years for the contralateral breast cancer risk model without *BRCA* gene mutation information.

The x-axis represents the predicted cumulative incidence of contralateral breast cancer at 10 years and the y-axis the observed cumulative incidence at 10 years. The black dots indicate the calibration for quartiles of predicted values. Vertical black bars indicate the 95% confidence intervals. The dashed gray line indicates perfect overall calibration. Each panel indicates a validation in one of the datasets. Panel A: Netherlands - BOSOM; Panel B: Netherlands - EMC; Panel C: Netherlands - NCR; Panel D: Europe – Scandinavia; Panel E: United States and Australia; Panel F: Europe – Other; Panel G: Europe – United Kingdom.

**
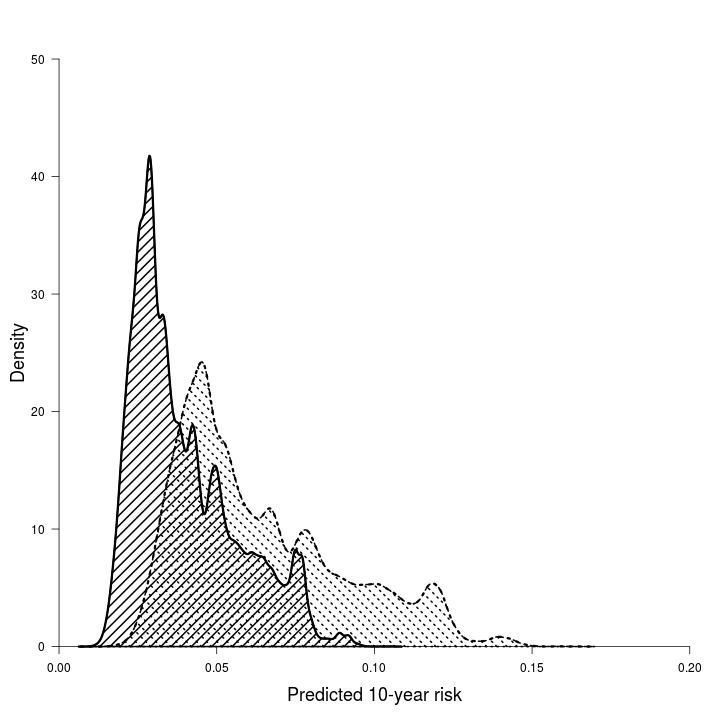
**

**Figure S8:** Density distribution of 10-year predicted absolute risk in patients with no family history (area with black lines) and patients with a family history (area with dashed lines).


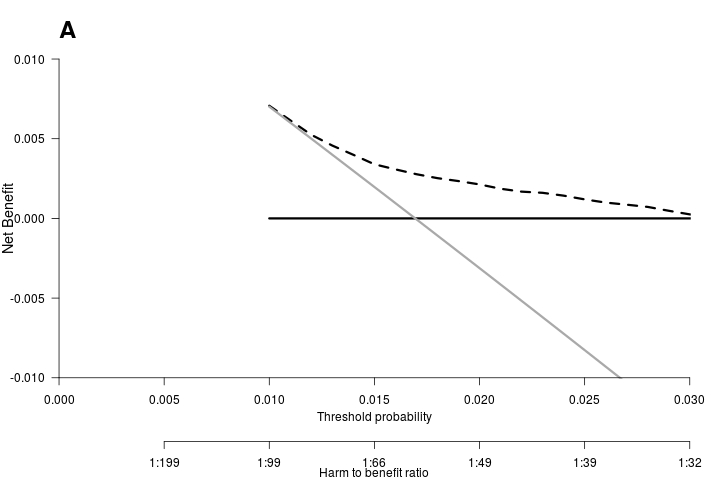

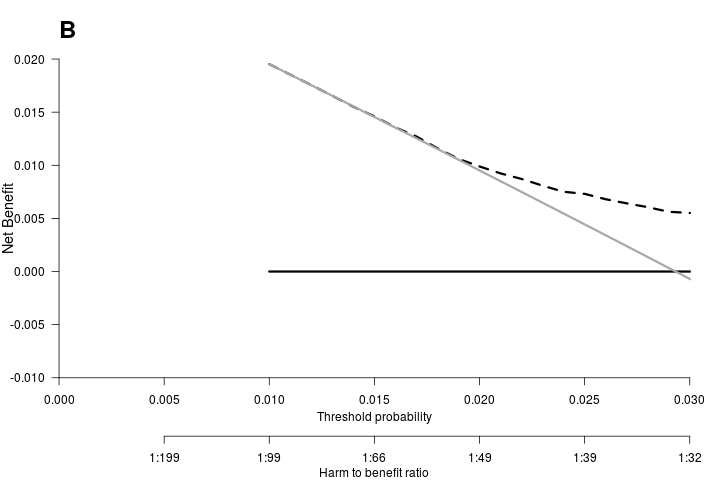


**Figure S9:** Decision curve analysis at 5 years for the contralateral breast cancer risk model without *BRCA* mutation information.

Panel A shows the decision curve to determine the net benefit of the estimated 5-year predicted contralateral breast cancer (CBC) cumulative incidence for patients without first-degree family history using the prediction model (dotted black line) compared to not treating any patients with contralateral preventive mastectomy (CPM) (black solid line). Panel B shows the decision curve to determine the net benefit of the estimated 5-year predicted CBC cumulative incidence for patients with first-degree family history of breast cancer using the prediction model (dotted black line) versus treating (or at least counseling) all patients (grey solid line). The y-axis measures net benefit, which is calculated by summing the benefits (true positives, i.e., patients with a CBC who needed a CPM) and subtracting the harms (false positives, i.e., patients with CPM who do not need it). The latter are weighted by a factor related to the relative harm of a non-prevented CBC versus an unnecessary CPM. The factor is derived from the threshold probability to develop a CBC at 5 years at which a patient would opt for CPM (e.g. 2.5%). The x-axis represents the threshold probability. Using a threshold probability of 2.5% implicitly means that CPM in 40 patients of whom one would develop a CBC if untreated is acceptable (39 unnecessary CPMs, harm to benefit ratio 1:39).


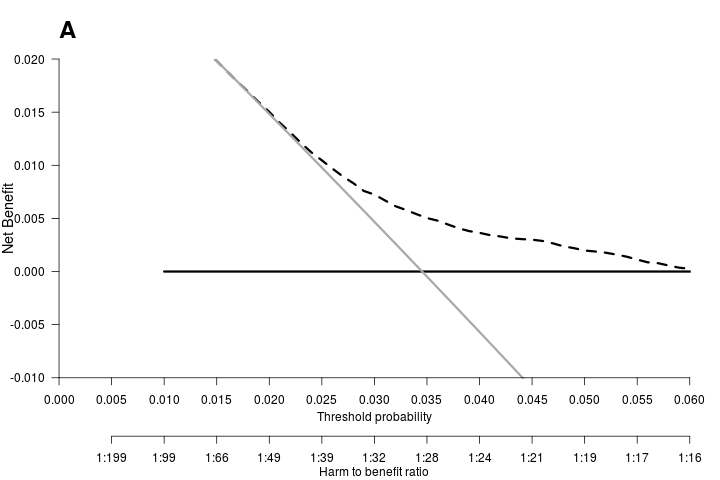

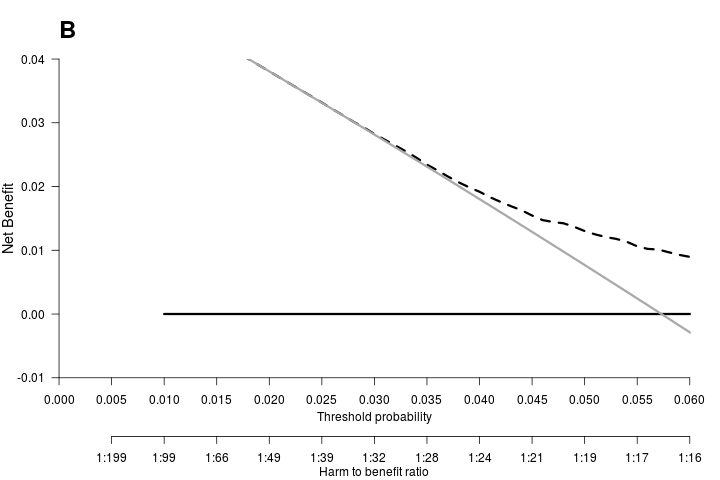


**Figure S10:** Decision curve analysis at 10 years for the contralateral breast cancer risk model without *BRCA* mutation information.

Panel A shows the decision curve to determine the net benefit of the estimated 10-year predicted contralateral breast cancer (CBC) cumulative incidence for patients without first-degree family history using the prediction model (dotted black line) compared to not treating any patients with contralateral preventive mastectomy (CPM) (black solid line). Panel B shows the decision curve to determine the net benefit of the estimated 10-year predicted CBC cumulative incidence for patients with first-degree family history using the prediction model (dotted black line) versus treating (or at least counseling) all patients (grey solid line). The y-axis measures net benefit, which is calculated by summing the benefits (true positives, i.e., patients with a CBC who needed a CPM) and subtracting the harms (false positives, i.e., patients with CPM who do not need it). The latter are weighted by a factor related to the relative harm of a non-prevented CBC versus an unnecessary CPM. The factor is derived from the threshold probability to develop a CBC at 10 years at which a patient would opt for CPM (e.g. 4.5%). The x-axis represents the threshold probability. Using a threshold probability of 4.5% implicitly means that CPM in 22 patients of whom one would develop a CBC if untreated is acceptable (21 unnecessary CPMs, harm to benefit ratio 1:21).


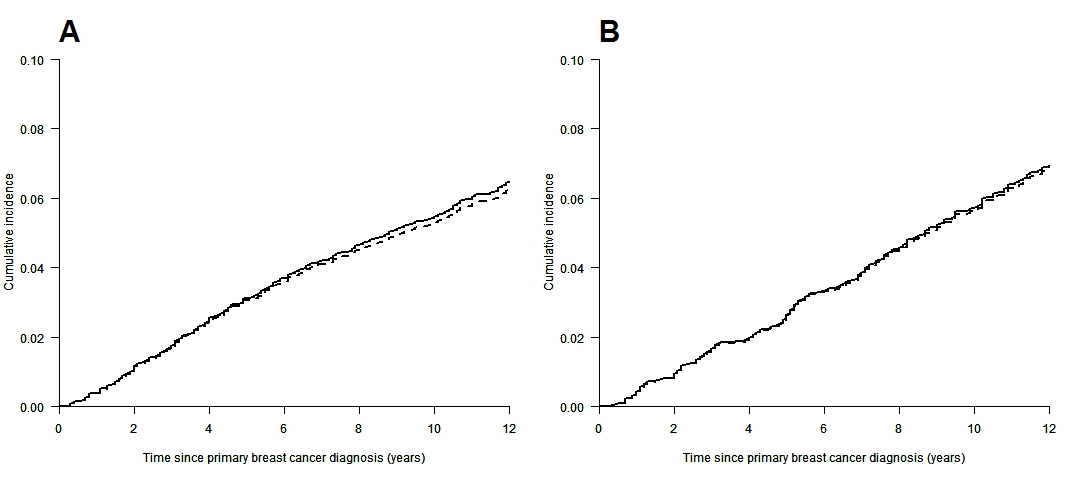


**Figure S11:** Assessment of inclusion of information of contralateral preventive mastectomy (CPM)**.**

Panel A shows the contralateral breast cancer cumulative incidence curve in the BOSOM dataset. Panel B shows the contralateral breast cancer cumulative incidence curve in the EMC dataset. The bolded black lines indicate the estimated cumulative incidence curve censoring patients with CPM at first primary breast cancer or during the follow-up. The dotted lines indicate the estimated cumulative incidence curve considering patients with CPM still at risk during the follow-up.
